# Nickel(II) com­plexes based on l-amino-acid-derived ligands: synthesis, characterization and study of the role of the supramolecular structure in carbon dioxide capture

**DOI:** 10.1107/S2052520620010008

**Published:** 2020-09-03

**Authors:** Andrea Rivas Marquina, Federico Movilla, Olga Carolina Sánchez Montilva, Eva Rentschler, Luca Carrella, Pablo Albores, Florencia Di Salvo

**Affiliations:** a Departamento de Química Inorgánica, Analítica y Química Física/INQUIMAE-CONICET, Facultad de Ciencias Exactas y Naturales, Universidad de Buenos Aires, Intendente Güiraldes 2160, Pabellón 2, Piso 3, Ciudad Universitaria, Ciudad de Buenos Aires, C1428EGA, Argentina; b Institute of Inorganic and Analytical Chemistry, Duesbergweg 10-12, Mainz, 55128, Germany

**Keywords:** amino acid, atmospheric CO_2_ uptake, supramolecular structure, nickel-carbonate system

## Abstract

The formation of the symmetrical μ_3_-carbonate-bridged self-assembled tri­nuclear Ni^II^ com­plex Na_2_{[Ni(LO)_2_(H_2_O)]_3_(μ_3_-CO_3_)} (LO is the carboxyl­ate anion of a l-tyrosine derivative), involves atmospheric CO_2_ uptake. The asym­metric unit of the com­plex com­prises an octahedral coordination for the Ni^II^ with two l-tyrosine-based ligands, a water molecule and one O atom of the carbonate bridge. The Ni_3_–μ_3_-CO_3_ core in this com­pound is the first reported of this kind according to the Cambridge Structural Database (CSD). The supramolecular structure is mainly sustained by hydrogen bonds developed by the phenolic functionality of the l-tyrosine moiety of one ligand and the carboxyl­ate group of a neighbouring ligand. The crystal packing is then characterized by three interpenetrated supramolecular helices associated with a diastereoisomer of the type *R*-^sup^ *P*, which is essential for the assembly process. Magnetic susceptibility and magnetization data support weak ferromagnetic exchange interactions within the novel Ni_3_–μ_3_-CO_3_ core. The Ni^II^ com­plex obtained under the same synthetic conditions but using the analogous ligand derived from the amino acid l-phenyl­alanine instead of l-tyrosine gives rise to to a mononuclear octahedral system. The results obtained for the different com­plexes demonstrate the role of the supramolecular structure regarding the CO_2_ uptake property for these Ni^II^–amino-acid-based systems.

## Abstract 2

Two l-amino-acid-based Ni^II^ com­plexes are reported; while the l-tyrosine derivative is a symmetrical μ_3_-carbonate-bridged self-assembled trinuclear Ni^II^ com­plex whose formation involves CO_2_ uptake, the l-phenyl­alanine analog is a mononuclear system which does not exhibit the same behaviour.

## 1. Introduction   

The fixation of CO_2_ by nickel ions in basic solutions and the subsequent generation of a carbonate com­plex is relevant to the bioinorganic, environmental, structural and materials chemistry fields. The metallocarbonate system has been studied extensively due to its central role in the reversible hydration of CO_2_ and the dehydration of bicarbonate pro­cesses catalyzed by carbonic anhydrase (Bertini *et al.*, 1987 ▸; Christianson & Fierke, 1996 ▸). Thus, the syntheses of many metal–carbonate models have been performed with the idea of contributing to the elucidation of the mechanism related to the catalytic activity of this enzyme (Palmer & Van Eldik, 1983 ▸; Lipscomb & Sträter, 1996 ▸). There is also considerable interest in the fixation of CO_2_ as metal com­plexes in order to explore possible ways of mitigating global warming by providing sustainable sources of C-products of higher value (Liu *et al.*, 2015 ▸; Leitner, 1996 ▸; Belli *et al.*, 2003 ▸; Zevenhoven *et al.*, 2006 ▸; Dickie *et al.*, 2008 ▸).

**Figure 1:**
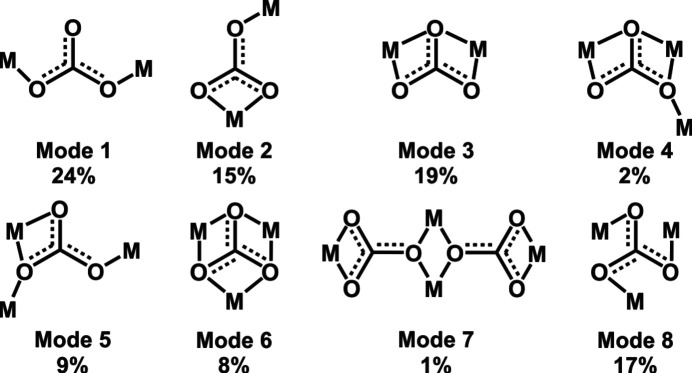
The coordination modes for CO_3_^2−^ acting as a bridging ligand in Ni^II^ carbonate-bridged systems, adapted from Anderson *et al.* (2009 ▸) using updated CSD results.

The carbonate ion is a very versatile ligand, since each O atom may act in a mono- or bidentate manner, and it is thus able to produce bi-, tri-, tetra-, penta- and hexanuclear coordination systems. This characteristic makes the carbonate ligand an interesting source for the design and synthesis of structurally rich com­plexes. The incorporation of more than one carbonate or in fact other bridging ligands can generate polynuclear com­plexes or even clusters which exhibit remark­able magnetostructural properties and, consequently, potential applications (Halmann, 2018 ▸). Considering that the three carbonate O atoms can participate in coordination to a metal centre and that each can exhibit a maximum of two bonds, seven different coordination modes are proposed. An additional mode, which is in fact a combination of two different modes, is observed in the Cambridge Structural Database [CSD; Groom *et al.*, 2016 ▸; representative examples of structures discussed in the text are identified by their CSD refcodes and a list of the associated references is available as supporting information] (Fig. 1 ▸, Mode 7; Anderson *et al.*, 2009 ▸) giving a total of eight modes (Fig. 1 ▸). Despite the versatility of the carbonate bridge, the majority of reported examples are Cu^II^-based com­plexes (see supporting information). An updated descriptive statistical analysis using the hits obtained from the CSD showed that every coordination mode is not equally found, modes 1 and 3 being the most common having 24 and 19% of the total, respectively (Fig. 1 ▸ and supporting information). They are followed by mode 8 (bridging μ_3_-carbonate) with 17% and mode 2 with 15% of the total. The remaining modes showed less than 10% of occurrence. For copper systems, the most frequently observed mode is mode 8 (supporting information).

Aromatic amino acids [tryptophan (Trp), tyrosine (Tyr) and phenyl­alanine (Phe)] are related to brain function. Several studies correlated the self-assembly of Phe with phenyl­ketonuria and β-amyloid-based neurodegenerative conditions, such as Alzheimer’s and Huntington’s diseases (Perween *et al.*, 2013 ▸; Adler-Abramovich *et al.*, 2012 ▸; Singh *et al.*, 2014 ▸; Do *et al.*, 2015 ▸) and atrial amyloidosis (Stefani, 2004 ▸). On the other hand, it is suggested also that the Tyr residue in amyloidogenic proteins acts as a key motif in the self-assembly process (Anjana *et al.*, 2012 ▸). In this context, a number of peptide-based building blocks related to these amino acids have been designed and developed to create supramolecular structures (Zhou *et al.*, 2017 ▸; Lee *et al.*, 2018 ▸). Moreover, the amino acids Phe and Tyr have been shown to be capable of self-assembling into supramolecular nanostructures (Adler-Abramovich *et al.*, 2012 ▸; Singh *et al.*, 2014 ▸; Ménard-Moyon *et al.*, 2015 ▸). Even though it is feasible for both Tyr and Phe-based fragments to be part of self-assembly processes, the structural difference associated with the phenolic OH group, as a side-chain functional group in Tyr, seems to be critical for the structural features and properties of the resultant entity. In this sense, the design and study of biomimetic analogous systems that help the understanding of the structural differences and properties among them could be considered important.

In our group, we investigated the synthesis of com­pounds derived from amino acids and explored their use as ligands for the construction of structurally diverse coordination com­plexes. In this work, we report the direct and reproducible synthesis of a trinuclear Ni^II^–carbonate com­plex exhibiting a μ_3_-CO_3_^2−^ coordination mode (Fig. 1 ▸, Mode 8). It was obtained under mild conditions using an N,O-based chiral ligand derived from the amino acid l-tyrosine (l-Tyr) and the aldehyde piperonal. To the best of our knowledge, this com­pound is the first amino-acid-based com­plex exhibiting the capability to self-assembly through CO_2_ fixation, a process that was not expected. Besides, the resultant symmetrical Ni_3_–μ_3_-CO_3_ core would be the first reported of this kind. Although there are a few examples in the literature of Ni^II^–carbonate com­plexes containing Schiff base ligands (Mukherjee *et al.*, 2008 ▸; Schmitz *et al.*, 2016 ▸), none of them are related to the amino-acid-based skeleton or the carbonate-bridging mode exhibited in our com­plex. Besides, overall, there is a limited number of examples of Ni^II^ com­plexes bearing l-Tyr or l-Tyr-based ligands (Groom *et al.*, 2016 ▸). On the other hand, the Ni^II^ com­plex synthesized using an analogous ligand obtained from l-phenyl­alanine (l-Phe) instead of l-Tyr and the same aldehyde produced an octahedral mononuclear com­pound. This information suggests that the functionalities of the ligands could be responsible for their structural features and behaviour against CO_2_ uptake which is, therefore, related to the supramolecular properties that sustain the self-assembly process. These easy-to-synthesize complexes could be con­sidered as a simple example to illustrate self-assembly properties along Tyr and Phe analog systems which have a close relationship with many cases in biological systems. Finally, the magnetic behaviour of the trinuclear Ni^II^ l-Tyr-based com­plex is also described as a com­plement of the study of the structural, supramolecular and physical properties.

## 2. Materials and methods   

### 2.1. General considerations   

UV–Vis spectra were recorded using a Hewlett–Packard 8453 diode array spectrometer in 10 mm optical path quartz cuvettes. Elemental analysis was carried out in a Carlo Erba CHNS EA-1108 microanalyzer using atropine as the standard. Mass spectra were recorded on a Xevo G2S Q-TOF (Waters Corporation) instrument using an electrospray ionization source and a quadrupole time-of-flight analyzer in methanol as the solvent. Differential scanning calorimetric (DSC) studies were performed in a Shimatzu DSC-50 with an aluminium pan under an N_2_ atmosphere. Thermogravimetric analysis (TGA) was performed in a Shimatzu TGA-50 with an aluminium pan. NMR spectra were recorded using a Bruker AM500 equipped with a broadband probe. ^1^H shifts are reported relative to protic solvent in these solvents. ^13^C shifts are reported relative to DMSO-*d*_6_ (δ) 39.52 ppm. IR spectra were recorded using a Nicolet Avatar 320 FT–IR spectrometer with a Spectra Tech cell for KBr pellets. Light micrographs using polarized light microscopy (PLM) were taken with a Nikon SMZ-745 T stereoscopic trinocular micro­scope that includes a Nikon Ni-150 lighting system. Images were processed using the program *Micrometrics SE Premium*. Scanning electron microscopy (SEM) images were produced using a Carl Zeiss NTS SUPRA 40. All samples were purified and dried before being placed over carbon tape strands in aluminium pin stubs.

### 2.2. Materials and general procedures   

All chemicals and solvents used for synthesis were obtained from commercial sources and were used as received, without further purification. Methanol was distilled before use. All reactions were carried out under aerobic conditions. The ligands **1**–**3** were synthesized following previously reported procedures for similar com­pounds with some modifications (Singh *et al.*, 2017 ▸; Kumar *et al.*, 2015 ▸).

### 2.3. Synthesis of the ligands   

#### 2.3.1. Ligand **1**   

To a solution of l-Tyr (110 mg, 0.6 mmol) and KOH (34 mg, 0.6 mmol) dissolved in distilled methanol (2.5 ml) were added piperonal (100 mg, 0.7 mmol) dissolved in of distilled methanol (0.5 ml). The resulting mixture was refluxed for 4 h. The yellow reaction mixture was then brought to room temperature and NaBH_4_ (46 mg, 1.2 mmol) was added under stirring at the same temperature. The resulting solution was refluxed for 20 min until the colour disappeared. The pH of the solution was adjusted to 5 using hydro­chloric acid and was then stirred for 1 h. The obtained white precipitate (**1**) was filtered off, washed with water and methanol, and dried under vacuum (yield: 150 mg, 78.4%). EI/MS: *m*/*z* calculated for [*M*]^+^ 315, found 315, other signals: 270, 208, 135, 107, 77, 51. CHN elemental analysis for C_17_H_17_NO_5_ calculated (%): C 64.8, N 4.4, H 5.4; found: C 64.3, N 4.4, H 5.7. NMR ^1^H (500 MHz, DMSO-*d*_6_): δ 7.04–6.95 (*d*, *J* = 8.4 Hz, 2H), 6.86–6.78 (*m*, 2H), 6.74–6.67 (*d*, *J* = 7.7 Hz, 1H), 6.69–6.59 (*d*, *J* = 8.4 Hz, 2H), 6.04–5.93 (*s*, 2H), 3.73–3.65 (*d*, *J* = 13.4 Hz, 1H), 3.61–3.50 (*d*, *J* = 13.4 Hz, 1H), 3.26–3.19 (*t*, *J* = 6.6 Hz, 1H), 3.20–3.10 (*s*, 1H), 2.88–2.80 (*dd*, *J* = 13.8, 6.2 Hz, 1H), 2.79–2.69 (*m*, 1H). ^13^C NMR (126 MHz, DMSO-*d*_6_): δ 155.69, 130.23, 130.14, 121.52, 115.13, 114.80, 108.67, 107.84, 100.76, 62.32, 50.42, 37.40. Selected FT–IR peaks (KBr, cm^−1^): ν(N—H) = 3180.2, ν(C=O) = 1577.56. Selected UV–Vis bands (in H_2_O/NaOH solution, nm): 239, 287. Spectra are included in the supporting information.

#### 2.3.2. Ligand **2**   

Ligand **2** was prepared following the procedure described for the preparation of ligand **1**, except that 120 mg (0.7 mmol) of l-Phe were used instead of l-Tyr, and after the addition of NaBH_4_, the reaction mixture was stirred at room temperature and not under reflux (yield: 175.5 mg 80.3%). EI/MS: *m*/*z* calculated for [*M*]^+^ 299.0, found 299; other signals: 208, 135. CHN elemental analysis for C_17_H_17_NO_4_ calculated (%): C 68.2, N 4.7, H 5.7; found: C 67.4, N 4.7, H 5.7. NMR ^1^H (500 MHz, DMSO-*d*_6_): δ 7.27–7.18 (*m*, 12H), 6.80–6.78 (*m*, 2H), 6.69–6.67 (*m*, 1H), 5.97 (*m*, 2H), 3.70–3.67 (*m*), 3.58–3.53 (*m*), 3,27 (*d*), 2.97–2.93 (*m*), 2.85–2.82 (*m*). ^13^C NMR (126 MHz, DMSO-*d*_6_): δ 190.52, 147.10, 129.32, 127.89, 125.92, 121.28, 108.53, 107.80, 100.71, 62.32, 50.60, 38.51. Selected FT–IR peaks (KBr, cm^−1^): ν(O—H) = 3446.3, ν(C=O) = 1612.3. Selected UV–Vis bands (in H_2_O/NaOH solution, nm): 233, 284. Spectra are included in the supporting information.

### 2.4. Synthesis of the com­plexes   

#### 2.4.1. Synthesis of com­plex **1Ni** starting from Ni(NO_3_)_2_·6H_2_O   

In a 5 ml vial, Ni(NO_3_)_2_·6H_2_O (4.6 mg, 0.016 mmol) was dissolved in methanol (1 ml) and a solution of the sodium salt of ligand **1** was then added. The solution of the ligand was prepared by dissolving **1** (10 mg, 0.032 mmol) and NaOH (2.5 mg, 0.063 mmol) in methanol (1 ml). The resulting turquoise solution of the com­plex was stirred overnight and, after removing the cap of the vial and covering with parafilm foil pierced with small holes, it was opened to the air and left for slow evaporation of the solvent at room temperature. After *ca* 3 d, green prismatic single crystals (**1Ni-SC**) were obtained and preserved in their mother liquors. After removing the crystals from the solution and leaving them under atmospheric conditions, the quality of the crystals was clearly affected; opacity and some fractures were observed. A polycrystalline material (**1Ni-PM**) was obtained after careful removal of the remaining supernatant and leaving it open to the air to dry. When the resultant crystalline material was dried under vacuum, a light-green amorphous powder was obtained (**1Ni-A**). The single crystals and the polycrystalline material were characterized by single-crystal and powder XRD, UV–Vis and FT–IR spectroscopy, and the magnetic behaviour was studied by EPR and SQUID. The solvent contents in **1Ni-SC** and **1Ni-PM** were quantified by thermal techniques and elemental analysis (yield: 95%). CHNS elemental analysis (%) for **1Ni-SC** with the chemical formula Na_2_{[Ni(LO)_2_(H_2_O)]_3_CO_3_}·11H_2_O·16MeOH, C_119_H_188_N_6_Na_2_Ni_3_O_63_ (*M*_r_ = 2932.83, solvent confirmed by TGA) calculated (%): C 48.7, H 6.5, N 2.9; found: C 48.0, H 6.2, N 2.8. CHNS elemental analysis (%) for **1Ni-PM** with the chemical formula Na_2_{[Ni(LO)_2_(H_2_O)]_3_CO_3_}·6H_2_O·3MeOH, C_106_H_126_N_6_Na_2_Ni_3_O_45_ (*M*_r_ = 2426.23) calculated (%): C 52.5, H 5.2, N 3.5; found: C 52.0, H 4.9, N 4.0. Selected FT–IR peaks (KBr, cm^−1^): ν(O—H) = 3600–3300 (*s*), ν(N—H) = 3278 (*s*), ν(C=O) = 1608 (*s*), ν(C=C) = 1585 (*s*), ν(C—O) = 1515, 1444 (*s*), ν(C—H) = 1398, 1384 (*s*), ν(C—O) = 1243 (*m*), ν(C—O) = 931 (*m*). UV–Vis in methanol [λ_max_, nm (∊, l mol^−1^ cm^−1^)]: 624 (21), 382 (54), 286 (33153), 227 (81045). Spectra and additional figures are included in the supporting information.

#### 2.4.2. Synthesis of com­plex **1Ni** starting from other Ni^II^ salts   

The same procedure as described above was followed using other Ni^II^ salts: NiSO_4_·6H_2_O (4.2 mg, 0.016 mmol), NiCl_2_·6H_2_O (3.8 mg, 0.016 mmol) and NiAc_2_·4H_2_O (4.0 mg, 0.016 mmol). In all cases, the results were the same as described for the crystals obtained using Ni(NO_3_)_2_·6H_2_O, in agreement with the discussion developed in the corresponding section. The single crystals and crystalline material obtained using these salts were studied by FT–IR and powder and single-crystal XRD (see supporting information).

#### 2.4.3. Synthesis of com­plex **2Ni** starting form Ni(NO_3_)_2_·6H_2_O   

In a 5 ml vial, Ni(NO_3_)_2_·6H_2_O (4.9 mg, 0.017 mmol) was dissolved in methanol (1 ml) and a solution of the sodium salt of ligand **2** was then added. The solution of the ligand was prepared by dissolving **2** (10 mg, 0.033 mmol) and NaOH (2.7 mg, 0.066 mmol) in methanol (1 ml). The resulting turquoise solution was left unaltered in the same vial and, after covering with parafilm foil with small holes, it was opened to the air and left for slow evaporation of the solvent at room temperature. After *ca* 3 d, light-blue sphere-like crystalline aggregates (**2Ni-CA**) were obtained. The crystalline material was then washed with water and methanol, and air dried (yield: 40%). CHNS elemental analysis (%) for **2Ni-CA** with the chemical formula [Ni(LO)_2_(H_2_O)_2_]·2H_2_O·0.5MeOH or C_34_H_36_N_2_NiO_10_·2H_2_O·0.5MeOH (*M*_r_ = 743.4, solvent confirmed by TGA) calculated (%): C 55.7, H 5.7, N 3.8; found: C 55.3, H 5.0, N 3.8. Selected FT–IR peaks (KBr, cm^−1^): ν(O—H) = 3608 (*s*), ν(N—H) = 3268 (*s*), ν(C=O) = 1602 (*s*), ν(C=C) = 1490 (*s*), ν(C—O) = 1438 (*s*), ν(C—H) = 1403 (*s*), ν(C—O) = 1247 (*s*), ν(C—O) = 925 (*m*). UV–Vis in DMSO [λ_max_, nm (∊, l mol^−1^ cm^−1^)]: 622 (9), 379 (168). Details are included in the supporting information. Crystals of **2Ni** suitable for single-crystal studies (**2Ni-SC**) were obtained after slow evaporation of the solvent from a dimethylformamide (DMF) concentrated solution of the com­plex.

#### 2.4.4. Synthesis of com­plex **2Ni** starting from other Ni^II^ salts   

The same procedure as described above was followed using other Ni^II^ salts: NiSO_4_·6H_2_O (4.2 mg, 0.016 mmol), NiCl_2_·6H_2_O (3.8 mg, 0.016 mmol) and NiAc_2_·4H_2_O (4.0 mg, 0.016 mmol). In all cases, the results were the same as described above, *i.e.* light-blue sphere-like crystalline aggregates. The crystalline material obtained using the tested salts was studied by FT–IR, diffuse reflectance spectra, SEM and powder XRD (see supporting information).

### 2.5. Powder X-ray diffraction (PXRD) studies   

Data were recorded on a PANalytical Empyrean diffrac­tometer equipped with a 4 kW Cu *K*α sealed X-ray tube (generator power settings: 60 kV and 100 mA) and a PIXcel^3D^ area detector using parallel beam geometry (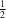
–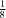
 mm slits, 15 mm incident mask). Samples were packed on a silicon monocrystal sample holder that was then placed on the sample holder attachment. For all PXRD experiments, data were collected over an angle range 5–90° with a scanning speed of 23 s per step and a 0.026° step.

### 2.6. Single-crystal X-ray diffraction (SC-XRD) measurements   

For the single crystals of **1Ni-SC** and **2Ni-SC**, good-quality results were obtained at the W01A-MX2 beamline of the Synchrotron National Laboratory (LNLS, Campinas, Brazil) using wavelengths λ = 0.79983 Å and 0.82610 Å respectively. Data were collected using a PILATUS2M area detector (Dectris). The measurements were performed at 100 K and data reduction was done using *MXCube* software (Gabadinho, 2010 ▸). Using *OLEX2* (Dolomanov *et al.*, 2009 ▸), the structures were solved by intrinsic phasing employing *ShelXT* (Sheldrick, 2015 ▸ *a*) and refined with the *ShelXL* (Sheldrick, 2015 ▸ *b*) package, using least-squares minimization. Non-H atoms were refined anisotropically. H atoms were mostly included at geometrically calculated positions with displacement parameters derived from the parent atoms. H atoms attached to the coordinated water molecules or to groups suitable for forming hydrogen bonds were located on Fourier maps, and refined using isotropic displacement parameters depending on the parent atoms.

For **1Ni-SC**, residual electron density associated with the solvent molecules was detected. Thus, data were treated with the SQUEEZE procedure (Spek, 2015 ▸) from *PLATON* (Spek, 2020 ▸). The volume occupied by the solvent was 3315 Å^3^ and the number of electrons per unit cell deduced by SQUEEZE was 854 electrons per void; these values could be interpretable as approximately 32 water and 29 methanol molecules. The presence of solvent in the structure is mostly in agreement with the results obtained by TGA/DSC measurements and experimental observations described previously. Additional crystallographic information and tables are included in the supporting information

For **2Ni-SC**, one of the coordinated water molecules (O10) is disordered over two sets of sites with occupancies of 0.49 and 0.51, and atom O1 of one of the carboxyl­ate groups of the ligands shows positional disorder over two sites with occupancies of 0.48 and 0.52 (Table S9 in the supporting information). Disorder was not modelled for the solvent molecules and is represented through large displacement ellipsoids. Additional crystallographic information and tables are included in the supporting information.

Crystals of com­plex **1Ni** obtained from different Ni^II^ salts were studied by single-crystal XRD using an Oxford Diffraction Gemini E lab diffractometer with Mo *K*α (λ = 0.71073 Å) radiation. After unit-cell determination it was ob­served that in all cases the obtained parameters and suggested space groups were com­parable with those of **1Ni-SC** synthesized using the nitrate salt. Due to the fact that experiments could be performed in *ca* 10 min, the crystal features and loss of crystallinity did not affect the data collection and quality (supporting information). Full data collection was performed at a synchrotron beamline as described.

### 2.7. Magnetic measurements   

Magnetic measurements were performed with a Quantum Design MPMS XL-7 SQUID magnetometer. All experimental magnetic data were corrected for the diamagnetism of the sample holders and of the constituent atoms (*M*_r_/2 × 10^−6^ cm^3^ mol^−1^ formula). DC measurements were conducted from 4 to 300 K at 1 kOe and at 4 K in the range 1–70 kOe. Variable-temperature X-band CW–EPR measurements were performed on a Bruker EMX-Plus spectrometer equipped with a nitro­gen continuous-flow cryostat (room temperature to 100 K) and a rectangular cavity with 100 kHz field modulation. The X-band CW–EPR spectra of the oriented single crystals or polycrystalline sample of **1Ni** were obtained as explained elsewhere (Schveigkardt *et al.*, 2002 ▸).

### 2.8. Quantum chemical calculations   

For com­putation of the exchange interaction *J* parameter, the *ORCA* (Neese, 2012 ▸) program package was employed. Single-point calculations for the high-spin (HS) and broken symmetry (BS) states at the X-ray geometry were carried out at the B3LYP level of density functional theory (DFT), employing the def2-TZVP Ahlrichs basis set for all atoms and taking advantage of the RI (Resolution of Identity) approximation. The SCF calculations were of the spin-polarized type and were tightly converged (10^−7^ Eh in energy, 10^−6^ in the density change and 10^−6^ in the maximum element of the DIIS error vector).

The methodology applied here relies on the BS formalism, originally developed by Noodleman for SCF methods (Noodleman, 1981 ▸), which involves a variational treatment within the restrictions of a single spin-unrestricted Slater determinant built upon using different orbitals for different spins. This approach was later applied within the framework of DFT (Baerends & Noodleman, 1984 ▸). The HS and BS energies were then combined to estimate the exchange coupling parameter *J* involved in the widely used Heisenberg–Dirac–van Vleck Hamiltonian. We used the method proposed by Ruiz and co-workers (Ruiz *et al.*, 1999 ▸, 2003 ▸), where the following equation is applied [equation (1)]: 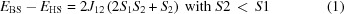 We have calculated the different spin topologies of BS nature by alternately flipping the spin on the different metal sites. The exchange coupling constants *J*_*i*_ are obtained after considering the individual pair-like com­ponent spin interactions involved in the description of the different BS states by solving a set of linear equations. We have also employed the BS-type spin unrestricted solutions after a corresponding orbital transformation (COT) as a means to visualize the interacting magnetic orbitals (Neese, 2004 ▸).

## 3. Result and discussion   

### 3.1. Synthesis of new amino-acid-based Ni^II^ com­plexes   

**Figure 2:**
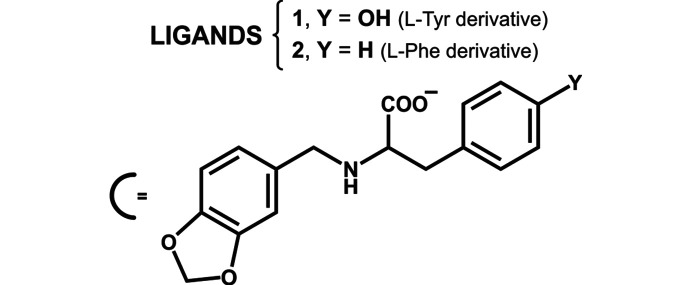
Ligands **1** and **2** obtained from the derivatization of the amino acids l-Tyr and l-Phe, respectively.

**Figure 3:**
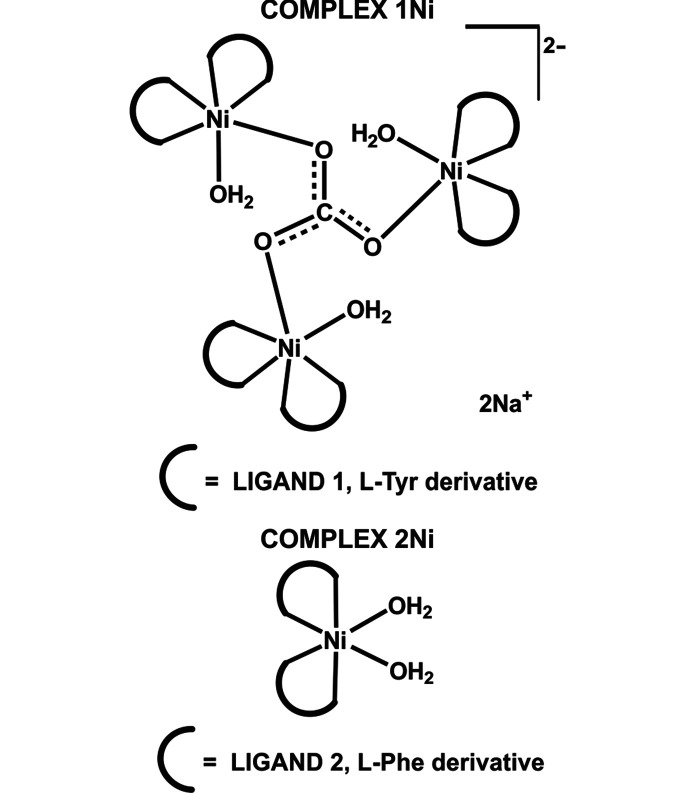
(Top) Com­plex **1Ni** obtained from the reaction of Ni^II^ salts with ligand **1** (l-Tyr derivative). (Bottom) Com­plex **2Ni** obtained when using ligand **2** (l-Phe derivative).

The molecules used as ligands (**1**–**3**) were obtained from the N-derivatization of l-α-amino acids through reaction with aldehydes and a subsequent reduction. Compounds **1** and **2** were synthesized using piperonal and the amino acids l-Tyr and l-Phe, respectively, and **3** used benzaldehyde and l-Tyr (Fig. 2 ▸ and supporting information). The reaction of the ligands with Ni^II^ salts gave coloured solids. Based on spectroscopic characterization and elemental analysis, the products were assigned as the corresponding coordination com­pounds but only after single-crystal X-ray diffraction (XRD) experiments were their structures unequivocally confirmed (Fig. 3 ▸). The results obtained for **1** and **2** are presented in detail, but those for **3** and the corresponding com­plex are included in order to study the effect of the piperonal moiety in the crystalline structure.

**Figure 4:**
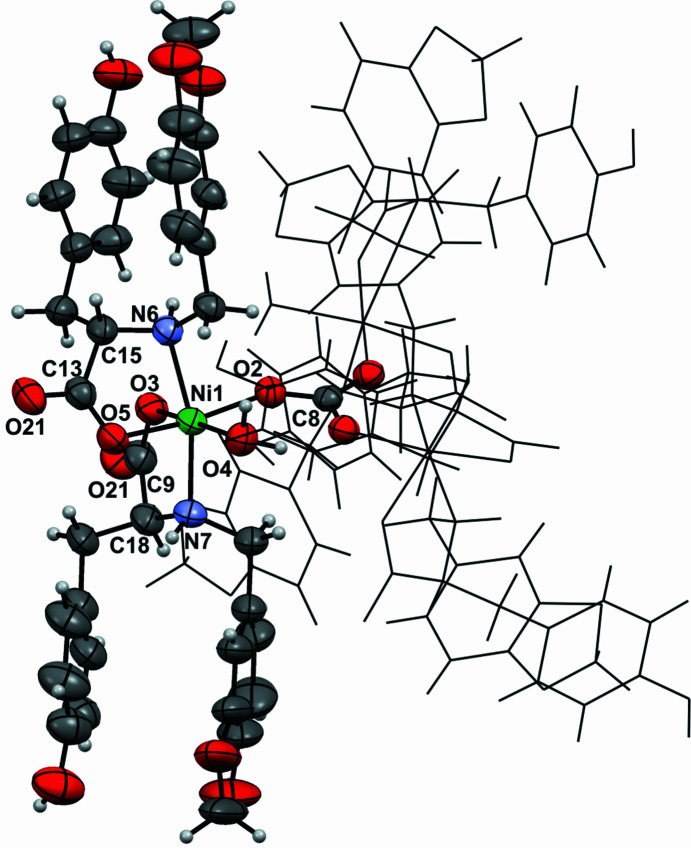
The structure of com­plex **1Ni** determined by single-crystal X-ray diffraction (**1Ni-SC**). For clarity, only the asymmetric unit and the com­plete carbonate ligand are shown as displacement ellipsoids at the 30% probability level; the rest of the com­plex is presented using grayscale wireframe. Sodium ions have been omitted.

Different Ni^II^ salts (acetate, nitrate, chloride and sulfate) were used as the starting materials for the synthesis of the com­plexes and, in all cases, the results were the same. This observation was a preliminary indication that the anions of the starting Ni^II^ salts were not part of the resulting com­plex. After mixing the starting materials in a 2:1 ligand-to-metal ion ratio, the resultant clear solutions were left for *ca* 12 h under stirring for ligand **1** but unaltered for **2**. After slow partial evaporation of the solvent, a coloured crystalline material was obtained in all cases. The reaction using ligand **1** gave large green–blue single crystals denoted **1Ni-SC**. Single-crystal XRD results showed a structure com­prising a trinuclear Ni^II^ com­plex with a carbonate ion as the bridging central ligand of the three metal ions (Figs. 4 ▸ and 5 ▸). The carbonate anion in the system is derived from the CO_2_ present in the atmosphere. Once it is spontaneously absorbed by the reaction mixture, it self-assembles into the trinuclear system. The com­plex is templated around the carbonate ligand, which is displayed in a triply bridging coordination mode, which is not the most frequent, as previously introduced.

The same synthetic procedure was followed using ligand **2**, except for the stirring, but instead of large single crystals, light-blue sphere-like crystalline aggregates constructed from nano- and microcrystals (**2Ni-CA**) were obtained (Figs. S9 and S10). They proved to be unsuitable for single-crystal XRD studies but, based on the spectroscopic characterization, the structure of **2Ni-CA** was suggested as an octahedral Ni^II^ com­plex bearing two deprotonated l-Phe-based ligands and two molecules of water. Crystalline material **2Ni-CA** was soluble only in DMF and dimethyl sulfoxide (DMSO). Concentrated DMF solutions of **2Ni-CA** were left in a vial open to the atmosphere and, after about two months, larger light-blue–green crystals were observed (denoted **2Ni-SC**). Single-crystal XRD experiments confirmed the previously proposed structure (Figs. 3 ▸ and 6 ▸). Taking into account that the synthetic procedures were very similar, the solubility and the coordination sites provided by both ligands were also equivalent, *a priori* it was suggested that a possible source for the different structural features and the property regarding the CO_2_ uptake could be directly associated with the structural variance provided by the phenol group of the Tyr residue and the supramolecular structure developed by each system.

### 3.2. Molecular and supramolecular structure of the trinuclear nickel–carbonate system **1Ni**   

**Figure 5:**
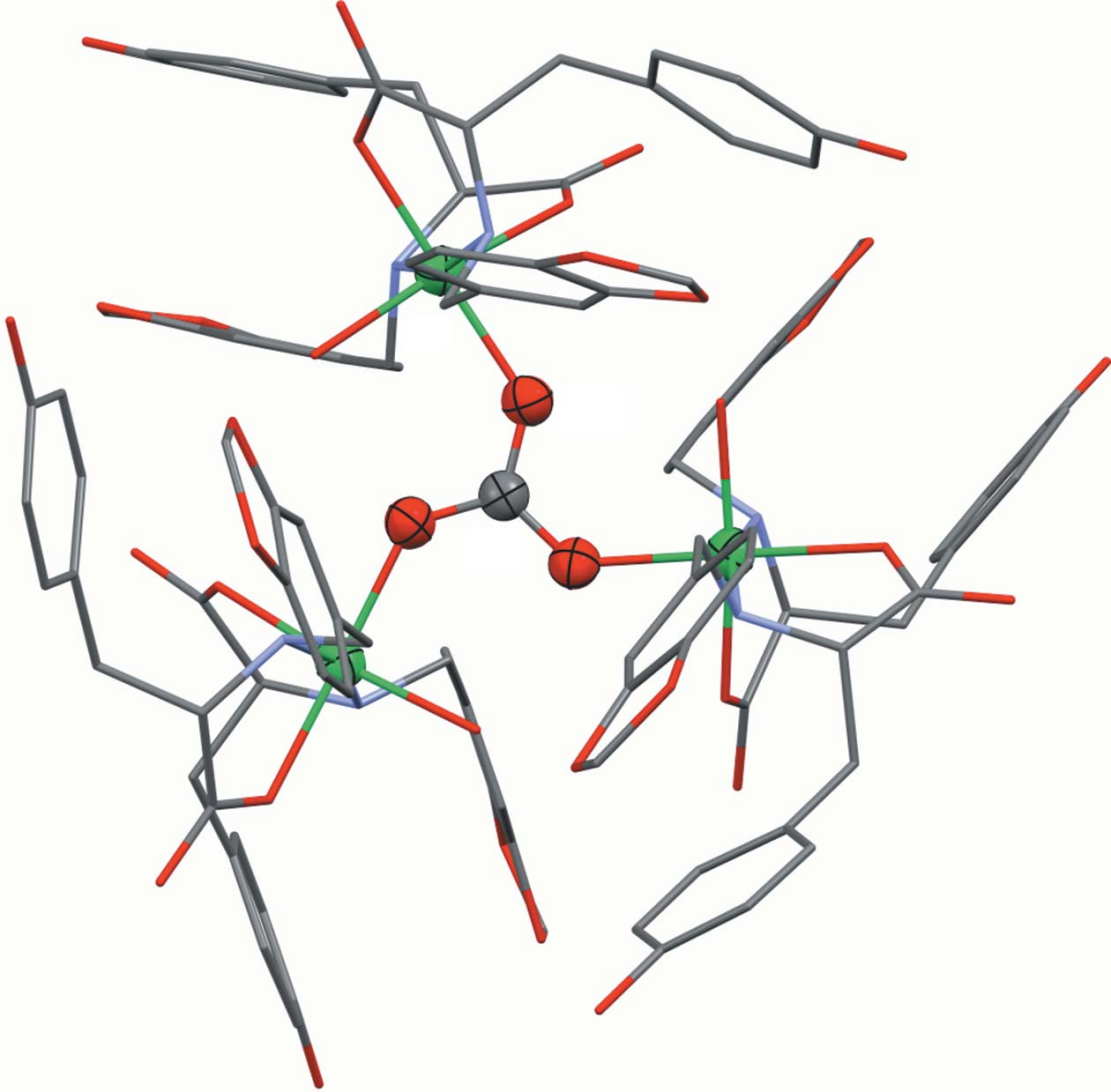
View of the trinuclear com­plex along the crystallographic *ab* plane, showing the central carbonate ligand with displacement ellipsoids at the 50% probability level and the rest of the atoms using a stick model. Colour code: O atoms red, C gray, N violet–blue and Ni green. H atoms, Na ions and disorder have not been included for clarity.

Even though crystals of the l-Tyr-based com­plex **1Ni-SC** were of good quality, single-crystal XRD studies were difficult to perform because of the large amount of solvent included in the structure. Once the crystals had been removed from the mother liquor, they had to be frozen or embedded in oil to prevent solvent loss from the structure and thus alteration of the crystallinity. Suitable measurements were nevertheless obtained using a Synchrotron beamline and a temperature of 100 K. Complex **1Ni** crystallized in the noncentrosymmetric space group *R*3 with a hexagonal unit cell [*a* = *b* = 20.020 (2), *c* = 31.13 (1) Å]. Three trinuclear com­plexes are observed per unit cell and there was a solvent-accessible volume of 3315 Å^3^ per unit cell, representing about the 30% of the total. The structure of the com­plex showed an unusual symmetry dictated by the μ_3_-CO_3_^2−^ ligand acting in a tridentate bridging mode with a *C*_3_ rotational axis located at the C atom. Each Ni^II^ centre possess a distorted octahedral geometry; every metal ion bonds to an O atom of CO_3_^2−^, two l-Tyr derivatives acting as bidentate chelating ligands binding through the carboxyl­ate group in a κ*O* mode and the secondary amine group, and finally a water molecule to com­plete the sixth position (Fig. 5 ▸). The total charge of the com­plex was determined as −2 and thus two sodium ions, coming from the NaOH used to generate the salt of the ligands, were also present in the structure. The three Ni—O—C angles are about 132.7° and the Ni⋯Ni distances are 5.332 (1) Å. The arrangement adopted by the carbonate ligand and the three Ni centres is planar and shows an average O—C—O bond angle of 119.99°. Crystallographic tables and com­plementary analysis are included in the supporting information.

Although the CO_2_ fixation and subsequent incorporation in the crystal structure as a carbonate ligand has been documented before for a few systems (Groom *et al.*, 2016 ▸), none of them were constructed using amino acids or their derivatives as ligands. What is more, the examples which are most closely related to the present com­plex are scarce. The results obtained after searching the CSD (Groom *et al.*, 2016 ▸) for polynuclear com­plexes having a CO_3_^2−^ ligand acting in a central bridging manner bonded to at least a minimum of three Ni^II^ centres, showed just 11 hits. None contain amino-acid-based molecules as part of their structure and only one includes an *N*,*O*-type ligand, but the N atom is part of a pyridine ring (Graham *et al.*, 2000 ▸). Only four hits correspond to com­plexes showing the μ_3_-CO_3_^2−^ arrangement of mode 8 as in **1Ni**, but they show more than three metal ions, confirming what was stated regarding the peculiarity of this binding mode (Fig. 1 ▸ and supporting information). When analyzing the structures of these com­plexes in detail, it is observed that all of them bear the same TMEDA (*N*,*N*,*N*′,*N*′-tetra­methyl­ethane-1,2-di­amine) ligand as a multi-chelating moiety which indeed provides extra stability (Fig. S4; Anderson *et al.*, 2009 ▸; Mustapha *et al.*, 2008 ▸; Miyamoto *et al.*, 2008 ▸). Another aspect of note is that **1Ni** is the only metallocarbonate system showing a symmetrical triangular array of a total of three nickel ions bearing a central μ_3_-CO_3_^2−^ bridge. Only five structures match with the core Ni_3_–carbonate, but with other coordination modes, making our com­plex the first of its kind (Fig. S5; Groom *et al.*, 2016 ▸). Regarding this type of symmetrical arrangement, Zn_3_ (Mohapatra *et al.*, 2019 ▸) and Cu_3_ (Mukherjee *et al.*, 2008 ▸; Kolks *et al.*, 1980 ▸; Escuer *et al.*, 1996 ▸) systems seem to be the most frequent and, concordantly, their structural and magnetic properties (just for copper) have been extensively studied (supporting information). When focusing on the amino acids, according to the CSD (Groom *et al.*, 2016 ▸), there are ten Ni^II^ com­plexes with unsubstituted l-Tyr and eight with l-Phe. In those com­pounds, the amino acids show typical chelating coordination *via* the N atom of the amino group and one of the O atoms of the carboxyl­ate group, as in **1Ni-SC** and **2Ni-SC**. Even though some of these com­plexes, as well as **2Ni**, were obtained under a similar synthetic procedure to **1Ni**, neither resulted in assemblies where CO_2_ uptake and its incorporation as carbonate are developed. All these observations reinforce the fact that com­plex **1Ni** is a rather unique system.

**Figure 7:**
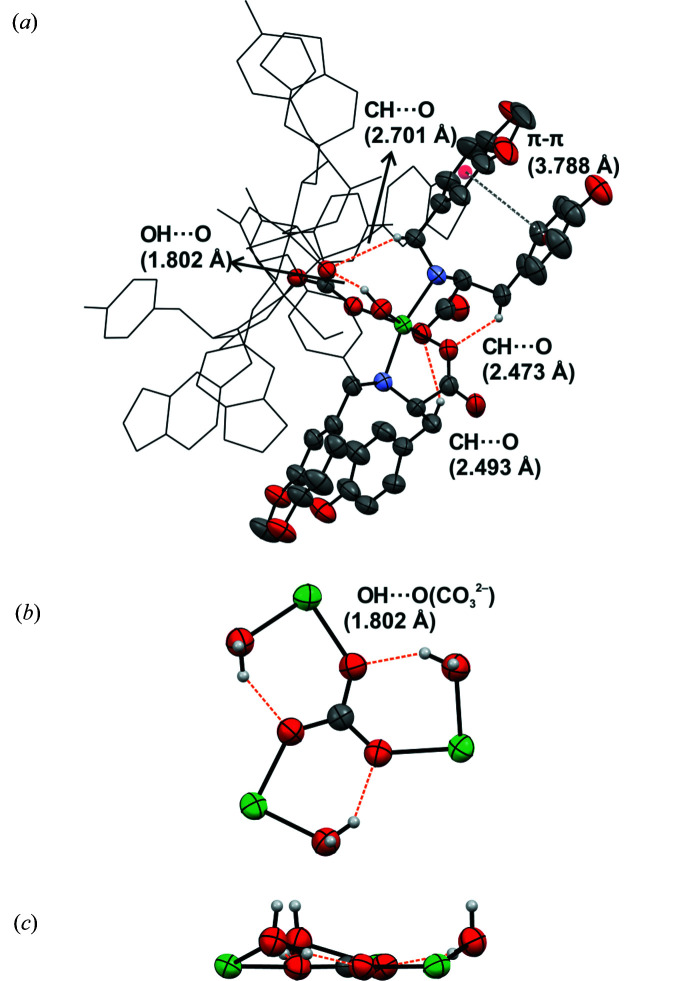
The main intramolecular contacts in **1Ni-SC**. For clarity, one asymmetric unit is displayed in displacement ellipsoid style and the rest in wireframe in gray. H atoms not involved in short contacts have been omitted. (*a*) π–π interactions and CH⋯O and OH⋯O contacts. (*b*, *c*) Hydrogen-bond sets between the coordinated water molecules and the carbonate bridging ligand along the *ab* and *bc* crystallographic planes, respectively.

The presence of COO^−^, OH and NH groups and the central carbonate ligand results in several intra- and intermolecular hydrogen bonds and other short contacts. Fig. 7 ▸ shows in detail the intramolecular interactions developed among the ligands and between them and CO_3_^2−^. It is interesting to point out that each O atom of the carbonate interacts with one of the H atoms of the water molecule coordinated to each Ni^II^ centre, giving a planar supramolecular moiety, which probably contributes to the stability of the trinuclear com­plex (Figs. 7 ▸ *b* and 7*c*). When the other four related structures based on the TMEDA ligand were inspected, a closely similar supramolecular hydrogen-bonded network around the central bridging ligand was observed. Taking into account that the related systems exhibiting mainly μ_5_- or μ_6_-binding modes for the carbonate are more frequent, it could be suggested that the stabilization of the structures showing μ_3_-CO_3_^2−^ could be associated mainly with the presence of other short contacts, such as the mentioned hydrogen bonds, related to the central bridging ligand (Fig. S6).

**Figure 8:**
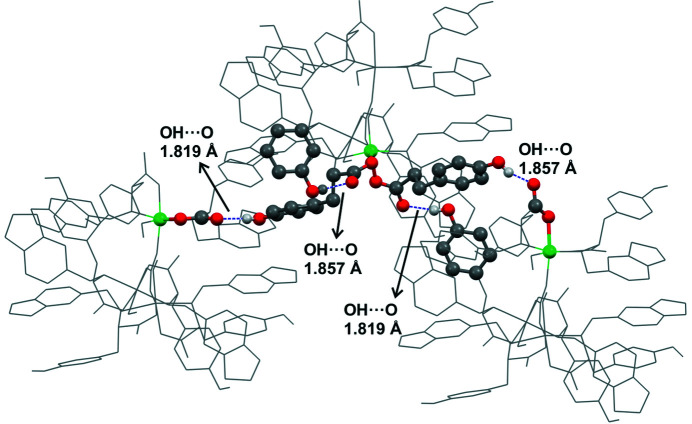
The OH⋯O contacts in **1Ni-SC** com­prising the carboxyl­ate and OH groups of l-Tyr. For clarity, only these moieties are depicted as coloured balls and ticks; the rest of the system is depicted in wireframe style in gray.

Several intermolecular interactions involving the ligands take place in **1Ni-SC** (Figs. 8 ▸ and S28). Among all the interactions, the hydrogen bonds developed by the phenolic functionality of the l-Tyr group of one ligand and the carboxyl­ate group of a neighbouring ligand could be considered as the driving force of the crystal packing [H⋯O distances are 1.819 and 1.857 Å]. The supramolecular structure of amino acids and their derivatives is usually described by the intermolecular electrostatic contacts defined by the amino/ammonium and carb­oxyl/carboxyl­ate groups, apart from other noncovalent interactions, such as hydrogen bonding, π–π stacking and hydro­phobic interactions (Bera *et al.*, 2018 ▸). The reason why the amino group in **1Ni-SC** does not participate in the supramolecular structure could be associated with the fact that the N-atom lone electron pair is mainly com­promised in the coordination bond and also steric effects. Looking closely to the structure of **1Ni-SC**, it is observed that each of the two ligands of each nickel centre of the trimeric unit shows a different hydrogen-bond network. In one of them, the carboxyl­ate and phenol OH groups interact with the corresponding hydrogen-bond donor and acceptor, respectively, of two molecules coordinated to two different nickel ions, but belonging to the same trinuclear com­plex. The other ligand of the same centre interacts with two molecules belonging to two different trinuclear units (Figs. 8 ▸ and 9 ▸). These networks give rise to the development of three different graph sets, but all of them are of the type 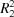
(27) (Fig. S30).

**Figure 9:**
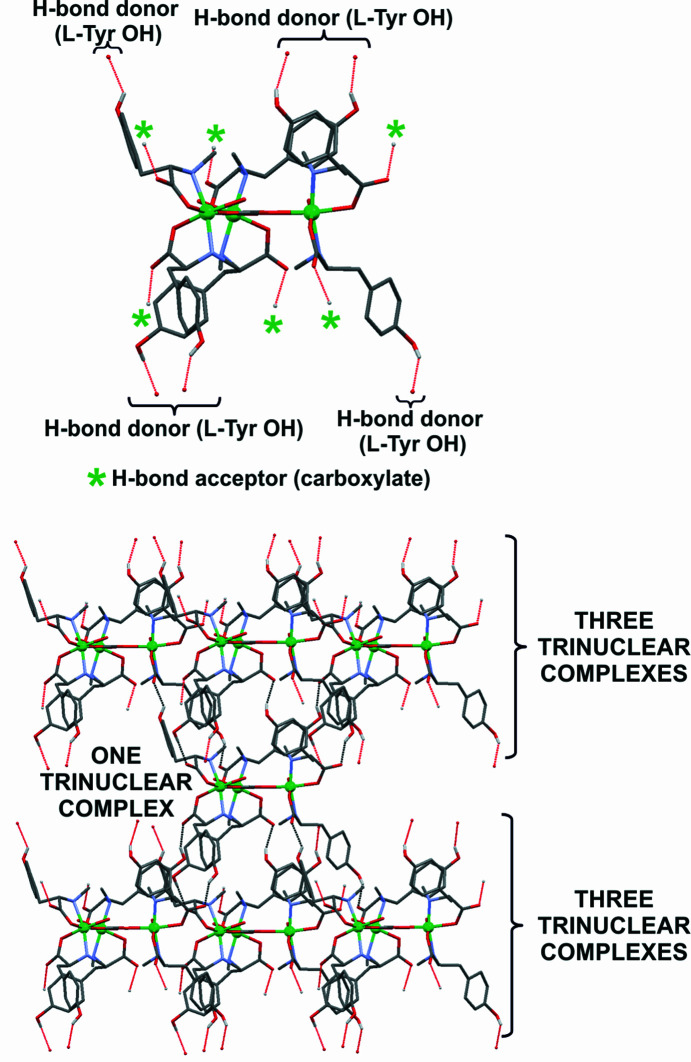
(Top) A trimeric unit with the hanging contacts (red dashed lines) starting from the O—H and COO^−^ hydrogen-bond donors and acceptors. (Bottom) The extended supramolecular structure, showing three trinuclear com­plexes above and below the starting unit; the expanded contacts are indicated in gray and the hanging contact is in red. In the images, the counter-ions, the piperonal moieties and H atoms which do not participate in the short contacts have been omitted for clarity.

Considering the trinuclear com­plex as a unit, an analysis shows that each of them interacts with another six units through the mentioned O—H⋯O contacts, three above and the other three below. The ligands of these six units then interact with others through the pendent hydrogen-bond acceptors and donors, resulting in three interpenetrated supramolecular helices. Another strategy to visualize the helices is through different chains that can be rationalized using the *Mercury* ‘Graph Set’ function (Macrae *et al.*, 2020 ▸). The voids containing the solvent molecules are located between these extended arrangements (Figs. 9 ▸ and 10 ▸).

**Figure 10:**
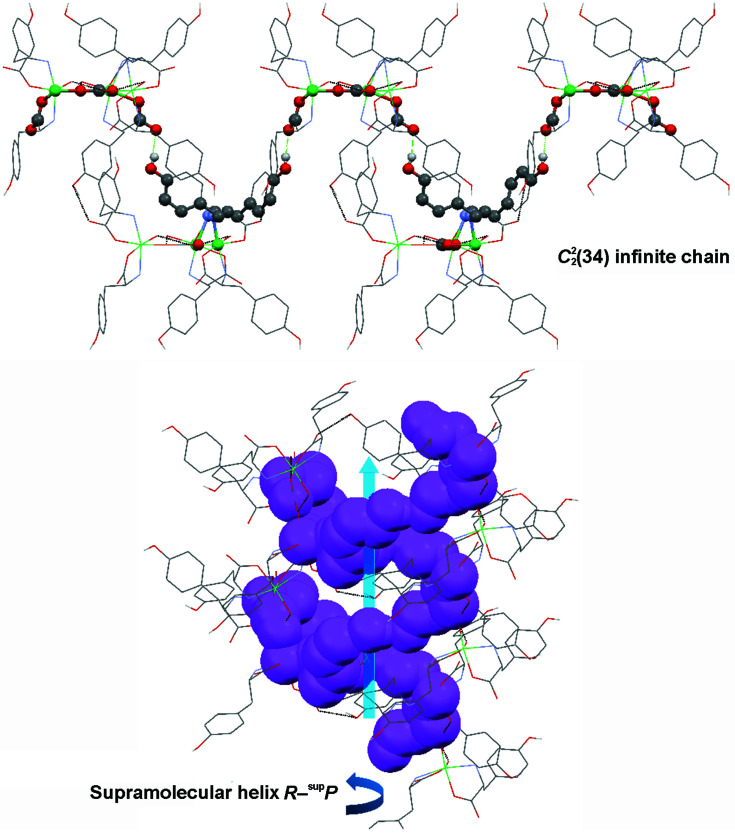
The extended 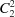
(34) infinite chain in **1Ni-SC**. (Top) The chain (in balls) along the crystallographic *bc* plane. (Bottom) View allowing visualization of the anticlockwise helix (space-filled pink spheres). In the images, the counter-ions, the piperonal moieties and H atoms which do not participate of the short contacts have been omitted for clarity.

On the other hand, since enantiomerically pure l-amino acids were used for the synthesis, ligands **1** and **2** retained their *S* configuration. Besides, considering each asymmetric unit of the com­plex as a *cis*-bis­(bidentate) octahedral system and the right-handed helix described by the ligands, they could be assigned with the Δ absolute configuration (Connelly *et al.*, 2005 ▸). The chiral nature of the trinuclear com­plex was then also expressed in the chiral space group *R*3 and with a Flack parameter of 0.035 (3). During the process of self-assembly in the crystalline solids of chiral molecules, supramolecular diastereoisomers can be generated. In the case of supramolecular helices, the resulting assembly can be described as *R* or *S*, depending on the configuration provided by the chiral molecule, followed by the term ^sup^ *P* if the produced helix is developed counterclockwise or ^sup^ *M* if it is clockwise (Tsang *et al.*, 2015 ▸; Sasaki *et al.*, 2013 ▸). Since com­plex **1Ni-SC** presents an *R* configuration (or Δ) and each helix has an anticlockwise orientation, the supramolecular structure can be clearly associated with a diastereoisomer of the type *R*-^sup^ *P* (Fig. 10 ▸).

### 3.3. Comparative structural analysis of the amino-acid-based Ni^II^ com­plexes   

**Figure 11:**
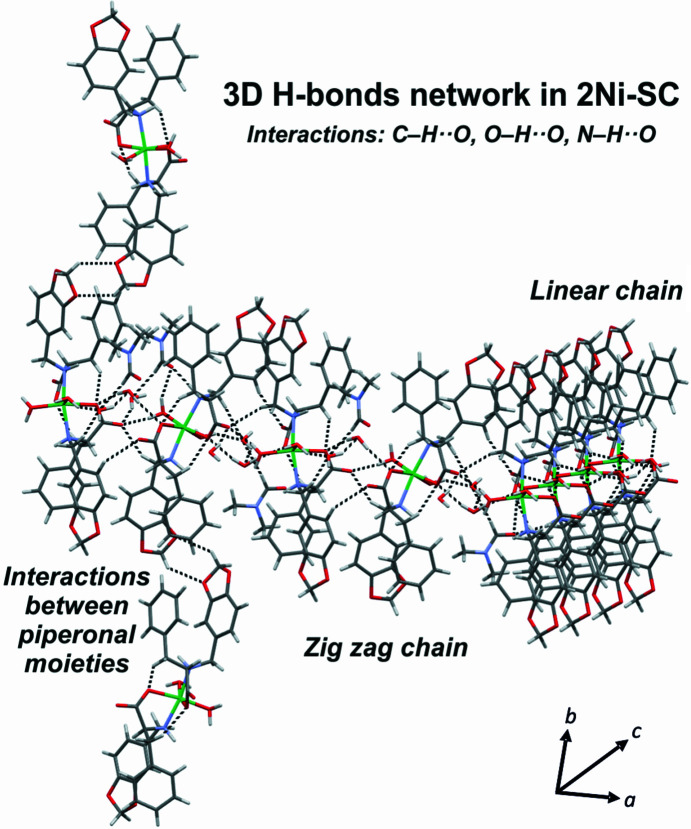
The hydrogen-bond 3D network in **2Ni-SC**.

The l-Phe-based com­plex **2Ni** was studied with the aim of exploring the role of the amino acid in CO_2_ uptake. Although differences in the supramolecular behaviour of l-Phe and l-Tyr are widely discussed in the literature (Adler-Abramovich *et al.*, 2012 ▸; Ménard-Moyon *et al.*, 2015 ▸), their implication in biologically relevant systems is a subject still under investigation (Yamagata *et al.*, 1998 ▸). As previously discussed, the central role of the phenol OH group in the molecular and supramolecular structure is clear. This functionality is provided by the l-Tyr moiety and is absent in the l-Phe derivative. Nevertheless, before the synthesis it was not clear how the behaviour of this system was going to affect CO_2_ uptake, since the synthetic procedure (identical for both com­plexes) using basic media could have been the driving force for carbonate inclusion in the structure, and not necessarily the supramolecular properties and the self-assembly. Then, as mentioned above, experimental data confirmed a mononuclear octahedral structure for the l-Phe derivative com­plex. This com­pound crystallized as a DMF and water solvate in the noncentrosymmetric space group *P*2_1_2_1_2, with an orthorhombic unit cell [*a* = 14.910 (3), *b* = 33.6200 (9), *c* = 7.370 (2) Å, see supporting information]. Solvent molecules are related to the com­plex through different hydrogen-bond motifs (Fig. S32). Each ligand is coordinated through the N atom of the amino group and one carboxyl­ate O atom, and they are not crystallographically equivalent. Two water molecules com­plete the coordination sphere in the equatorial positions, giving place to a *cis*-bis­(bidentate) octahedral system, as was observed for each asymmetric unit of the trinuclear com­plex **1Ni-SC**. The bond lengths and angles exhibited in **2Ni-SC** are similar to those observed for **1Ni-SC**. The configuration for **2Ni** corresponds also to the Δ enantiomer (Figs. 4 ▸ and 6 ▸). These results support the fact that the coordination environment for the metal centre can be considered as equivalent for both com­plexes and the main difference between them lies in the self-assembly process adopted in the l-Tyr com­plex due to carbon dioxide uptake. All of these originate from the presence of the phenolic OH group. The supramolecular structure observed for com­plex **2Ni** is com­pletely influenced by the presence of the solvent molecules because they act as excellent hydrogen-bond donors and acceptors. The crystal packing of **2Ni** describes a three-dimensional (3D) hydrogen-bond network sustained by interactions involving the ordinated water molecules, the amino and carboxyl­ate functionalities of the ligands, and the solvent molecules (Fig. 11 ▸ and Fig. S33 in the supporting information).

Finally, and in order to cover all the possible key factors acting in the supramolecular structure of trinuclear com­plex **1Ni**, the same reaction was performed but using ligand **3**, the l-Tyr-benzaldehyde analog (supporting information). The idea was to test the apparently negligible effect of the piperonal ring *versus* a phenyl group. The synthesis of com­plex **3Ni** gave rise to light-blue crystalline aggregates with low solubility and very similar features to what was observed for **2Ni** (Fig. S11). So far, single crystals of this com­plex have not been obtained and as a preliminary conclusion it could be inferred that the behaviour of this ligand is not equivalent to **1**. The piperonal moiety is involved in the supramolecular structure of com­plexes, not only in the structure of **1Ni**, but also in that of **2Ni**. Unfortunately, we cannot confirm our suggestion since we do not have single-crystal X-ray diffraction data for com­plex **3Ni**. Finally, it should be mentioned that even though the supramolecular architecture described in **1Ni** is mainly sustained by O—H⋯O=C hydrogen bonds involving the phenol and carboxyl­ate groups (Fig. 8 ▸), the O—CH_2_—O group of piperonal, through the development of C–H⋯O contacts, also influences the stability of the crystal packing.

### 3.4. Magnetic properties   

**Figure 12:**
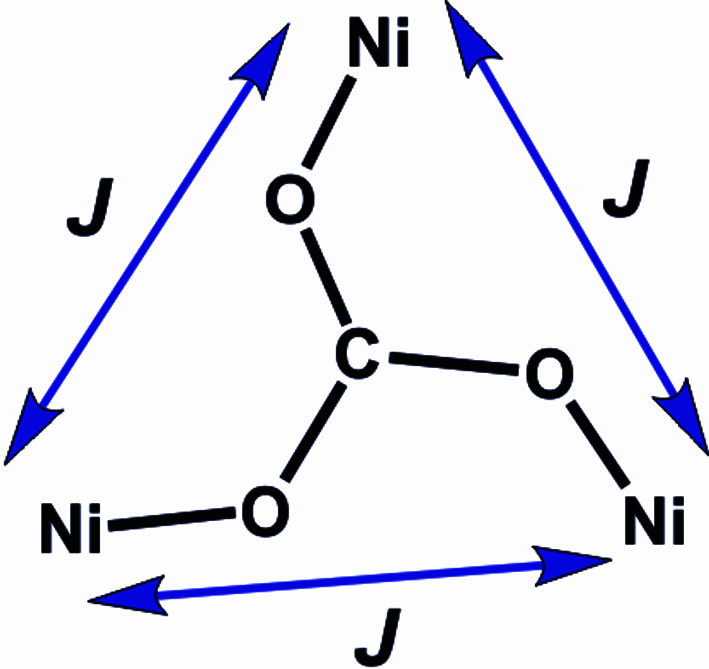
The pattern of exchange interactions in the **1Ni** com­plex.

The magnetic behaviour of the trinuclear Ni^II^ system was investigated by performing direct current (DC) magnetic susceptibility measurements on ground single crystals of com­pound **1Ni**, as well as magnetization measurements at 4 K (Fig. 12 ▸). The χ*T* value of 3.107 cm^3^ K mol^−1^ at room temperature is in good agreement with the expected value for three independent Ni^II^ ions of 3.00 cm^3^ K mol^−1^ (three *S* = 1 with *g* = 2; Kahn, 1993 ▸). With decreasing temperature, χ*T* remains almost constant until 50 K, when it increases sharply to a maximum value of 6.798 cm^3^ K mol^−1^ at *ca* 7 K; it finally drops to a value of 5.388 cm^3^ K mol^−1^ at 4 K. This behaviour suggests the presence of ferromagnetic exchange interactions within the Ni^II^ ions. In fact, the expected value for χ*T* for an isolated *S* = 3 ground state (arising from com­plete spin alignments due to ferromagnetic dominant exchange) is 6.00 cm^3^ K mol^−1^ (*g* = 2; Mohapatra *et al.*, 2019 ▸) and is in agreement with the observed value at the maximum of *χT* *versus* *T* data profile. The χ*T* lowering below 7 K is most probably attributable to the onset of zero-field splitting (ZFS) arising from local Ni^II^ ions. A magnetization plot at 4 K does not show saturation in agreement with ZFS onset, reaching a maximum value of 4.20 Nμ_B_ at 7 T.

In order to further understand the magnetic behaviour, we performed different experimental data fitting with the *PHI* package (Chilton *et al.*, 2013 ▸). The arrangement of the Ni^II^ ions with the unique carbonate bridge corresponds to a regular triangular array of sites with *S* = 1 (Fig. 12 ▸) due to the crystallographically imposed *C*_3_ axis.

**Figure 13:**
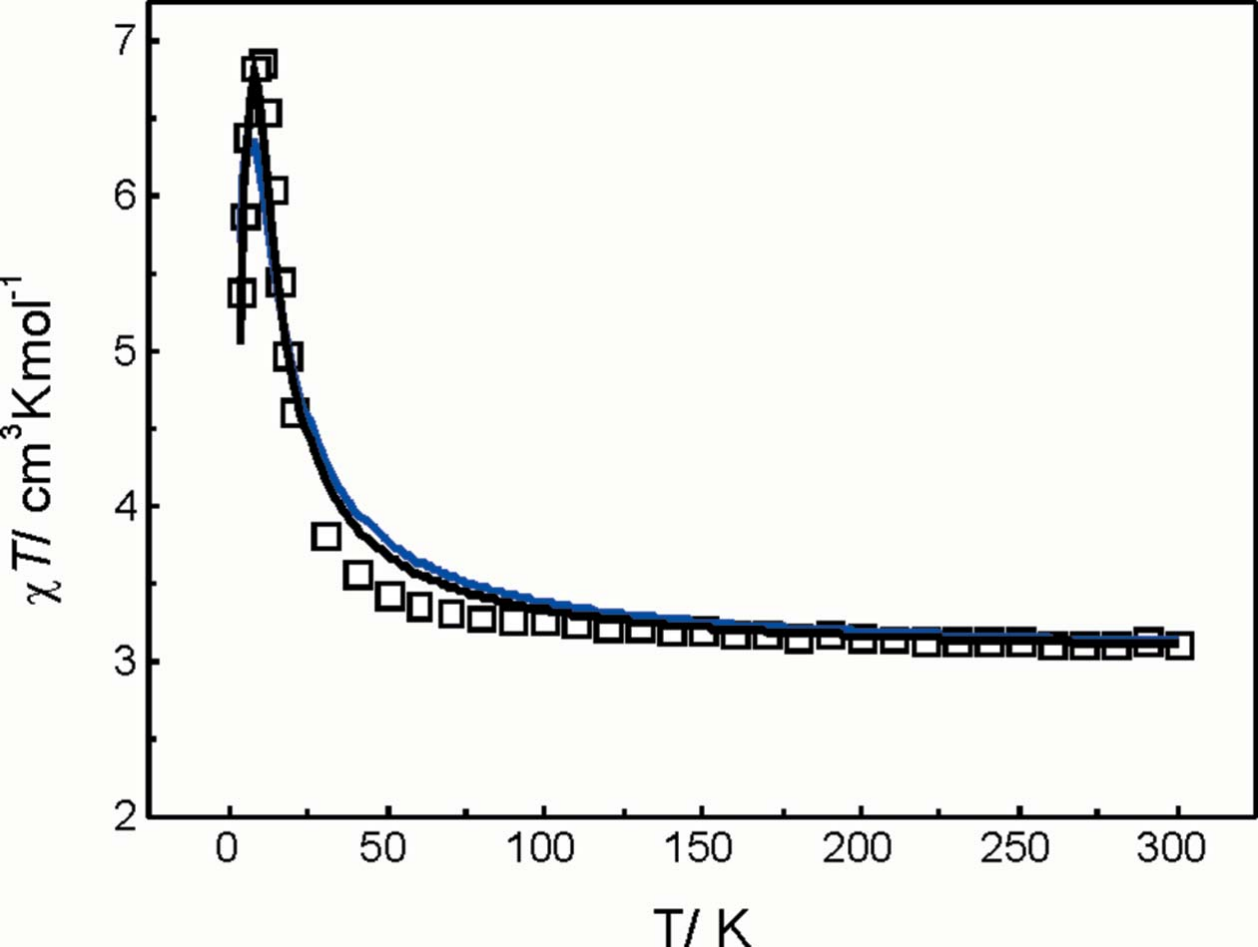
χ*T versus T* plot at 1 kOe external magnetic field of com­pound **1Ni**. Open symbols represent the experimental data, full lines represent the simulated data with best-fit parameters, black represents χ*T* data only and blue represents simultaneous χ*T* and M data.

This arrangement implies a unique exchange interaction parameter *J* for the three possible Ni^II^⋯Ni^II^ exchange pathways. Equation (2) shows the spin Hamiltonian suitable for this spin topology, including an axial local ZFS term on each equivalent Ni^II^ site. 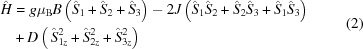 If the data fitting is performed employing only magnetic susceptibility data (χ*T* *versus* *T* plot), a satisfactory result can be obtained (Fig. 13 ▸) only if an intermolecular mean-field corrections is added (if not, low-temperature data cannot be reproduced), as shown in equation (3).
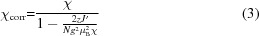



The low-temperature data fitting is impossible when removing either the ZFS or the *zJ*′ terms. The best fitting parameters obtained are: *g* = 2.0, *J* = 1.3 cm^−1^, *D* = 18.5 cm^−1^ and *zJ*′ = 0.46 cm^−1^. On the other hand, if a simultaneous fitting of magnetic susceptibility and magnetization data is performed, a poorer agreement is obtained for the *χT* *versus* *T* profile, than if only magnetic susceptibility is considered for data fitting (Fig. 13 ▸), it gives rise to a model which is in very very good agreement with the magnetization plot (Fig. 13 ▸). It does not happen if one tries to model both magnetic susceptibility and magnetization data simultaneously. Nevertheless, the best fitting parameters obtained within this approach are com­parable to those previously shown for the magnetic susceptibility only data fitting: *g* = 2.0; *J* = 2.6 cm^−1^; *D* = 16.1 cm^−1^ and *zJ*′= 0.18 cm^−1^.

**Figure 14:**
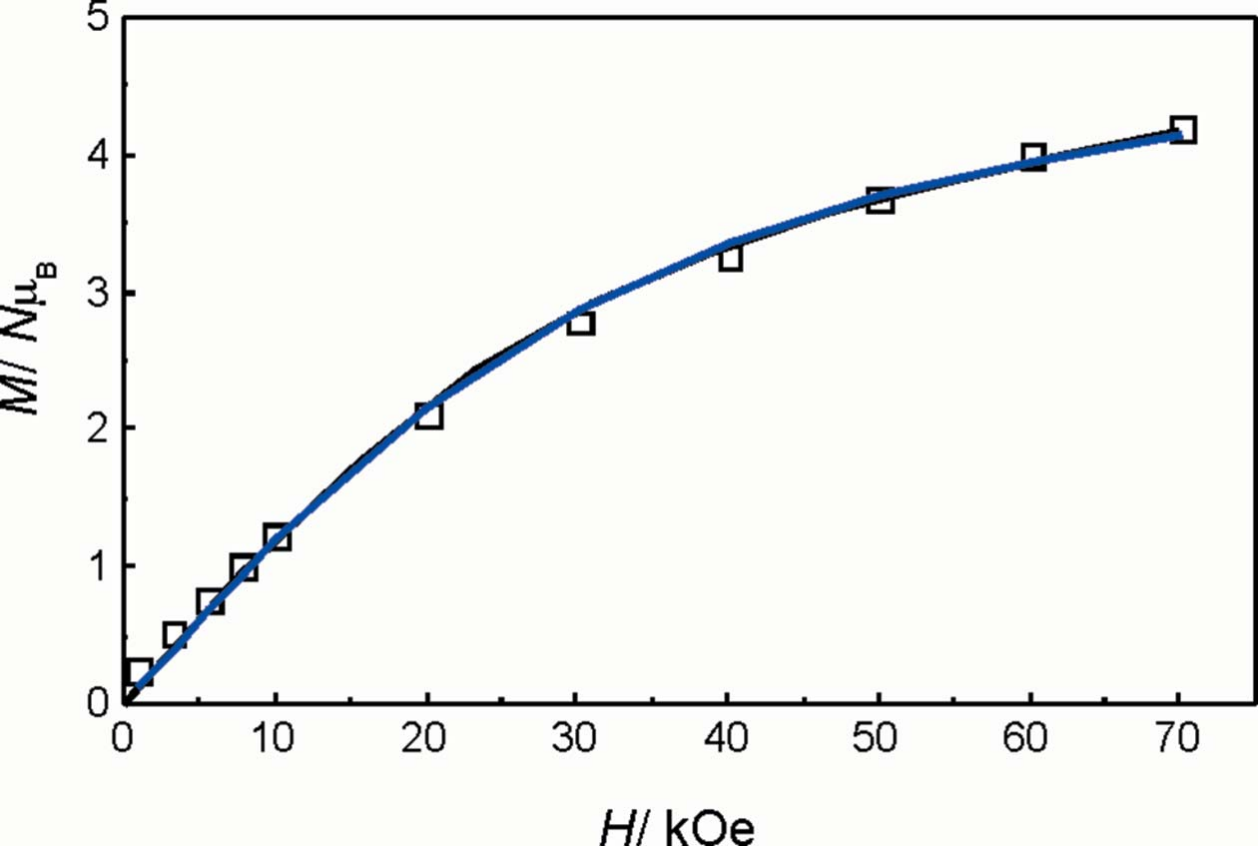
*M versus H* plot at 4 K of com­pound **1Ni**. Open symbols represent experimental data, full lines represent simulated data with best-fit parameters, black represents Hamiltonian equation (2) (three coupled *S* = 1) and blue represents Hamiltonian equation (4) (isolated *S* = 3).

Finally, even with a ZFS contribution larger than the exchange interaction, it is possible to fit the magnetization data at 4 K with an *S* = 3 isolated spin model having an axial ZFS contribution [Equation (4))], in agreement with the observed ferromagnetic *J* parameter for the carbonate-mediated Ni^II^⋯Ni^II^ exchange pathways (Fig. 14 ▸). The best fitting parameters in this case are: *g* = 2.17 and *D* = 13.9 cm^−1^. The simulated plot is indistinguishable from that obtained with Hamiltonian equation (2). 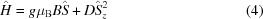



Regarding the parameters obtained from this DC magnetic data analysis, the dominant ZFS contribution is explained in terms of the small values of the exchange interaction, as the obtained values for the *D* parameter are frequently observed in Ni^II^ systems (Atanasov *et al.*, 2012 ▸). No reasonable agreement with the experimental data is reached if a negative *D* parameter is employed.

With respect to the found small ferromagnetic exchange coupling parameter, a precise determination of its value is not trivial for com­plex **1Ni**, as from magnetic susceptibility data fitting a strong correlation with the *zJ*′ parameter is observed (supporting information). Considering that for the mean-field approximation validity *J* > *zJ*′, a range of 1.0–2.5 cm^−1^ can be established for the *J* parameter with possible values of *zJ*′ in the range 0.2–0.5 cm^−1^. The existence of quite a strong intermolecular exchange interaction can be rationalized in terms of the one-dimensional nature of the supramolecular arrangement of the Ni_3_ com­plexes (*cf*. structural discussion).

Finally, the observed small ferromagnetic Ni^II^⋯Ni^II^ exchange interaction through the carbonate bridge deserves special consideration. To the best of our knowledge, there are no structurally characterized reported examples of trinuclear Ni^II^ com­plexes where a carbonate bridge adopts the μ_3_–*syn*–*anti* mode (Fig. 1 ▸, Mode 8) as the unique bridging ligand. In this sense, this becomes the first report of magnetic behaviour of this type for an Ni_3_-μ_3_-CO_3_ system. From studies of related Cu com­plexes, it has been stated that this bridging mode favours ferromagnetic Cu^II^⋯Cu^II^ interactions (Dussart *et al.*, 2002 ▸; Newell *et al.*, 2005 ▸; Martínez-Prieto *et al.*, 2013 ▸; Yoo & Lee, 2016 ▸; Jonasson *et al.*, 2018 ▸; Gerwien *et al.*, 2018 ▸). However, the existence of an additional magnetic orbital in Ni^II^ ions, together with low-symmetry environments, makes a direct com­parison unreliable. A few dinuclear Ni^II^ com­plexes bearing a carbonate bridge as the unique exchange pathway mediator can be found in the CSD (Groom *et al.*, 2016 ▸). All of them show a μ_2_–*anti*–*anti* bridging mode, and magnetic studies have been performed for only one of them, highlighting a small antiferromagnetic interaction (Dussart *et al.*, 2002 ▸). On the other hand, there are also a few reported Ni^II^ trinuclear com­plexes where the carbonate bridge appears as the unique ligand mediating the exchange interaction (Fig. S6; Dussart *et al.*, 2002 ▸; Newell *et al.*, 2005 ▸; Martínez-Prieto *et al.*, 2013 ▸; Yoo & Lee, 2016 ▸; Jonasson *et al.*, 2018 ▸; Gerwien *et al.*, 2018 ▸). In these systems, it is observed that the Ni^II^⋯Ni^II^ exchange interaction is through the Ni—O—C—O—Ni pathway and it is at most 10 cm^−1^, clearly weaker than the interactions through the Ni—O_carbonate_—Ni pathways (*e.g.* modes 2 to 7 in Fig. 1 ▸). These results agree with those observed for com­plex **1Ni**. However, predicting the nature (ferro or antiferro) of the exchange interaction in Ni^II^ carbonate-bridged com­plexes is still not obvious and no magneto-structural correlations are available.

As **1Ni** has an unprecedented carbonate-bridging mode for trinuclear com­plexes, we performed broken symmetry (BS) DFT calculations on the X-ray geometry in order to support the experimental magnetic data. Due to the *C*_3_ axis, the unique BS state (Fig. S35) was sufficient to obtain a quantum com­puted value for the Ni^II^⋯Ni^II^ exchange interaction. The obtained value for *J* of 0.19 cm^−1^ is in good agreement with the experimental results and supports the ferromagnetic nature of the exchange interaction. When looking at the magnetic orbitals, it can be observed that overlaps are negligible (Fig. S36), thus explaining the observed ferromagnetic exchange. More examples like this Ni_3_ carbonate-bridged com­plex are needed to further understand its role in the exchange interaction propagation.

Crystalline samples of **1Ni** were also investigated using X-band EPR spectroscopy. Neither the polycrystalline samples (**1Ni-PM**) nor the single-crystal samples of the trinuclear com­plex (**1Ni-SC**) measured in the range from ambient temperature to 100 K gave EPR signals. Hence, we suggest that com­plex **1Ni** is EPR silent under these experimental conditions, supporting the sizeable Ni^II^ local ZFS inferred from the magnetic susceptibility and magnetization data (Krzystek *et al.*, 2002 ▸).

## 4. Conclusions   

We observed that CO_2_ air fixation is a critical tool in the assembly of an l-Tyr-based trinuclear Ni^II^ com­plex. The higher nuclearity of this com­pound in com­parison with the mononuclear l-Phe analog is provided, on one hand, by the multiple bridging mode of the incorporated carbonate ligand and, on the other hand, by the identity of the amino acid. It is suggested also that the self-assembly process and the resulting structure is governed by the nature of the amino acid derivative through the supramolecular structure that is developed. The l-Tyr system showed a helix-like supramolecular assembly mainly sustained by hydrogen bonds provided by the carboxyl­ate and phenol functionalities. For the l-Phe analog com­plex, where such interactions cannot take place due to the absence of the phenol group, the supramolecular structure and thus the self-assembly followed a com­pletely different scheme. On the other hand, for the stabilization of the packing, the moiety provided by the aldehyde used for the synthesis of the ligands seems to also be important; in the absence of the piperonal skeleton in the com­plex obtained using a l-Tyr ligand derivatized with benzaldehyde, the results were com­pletely different. As seen in biological systems, strong differences in the supramolecular hierarchy are generated for different amino acids. In this sense, this work could be considered as a simple example to demonstrate the direct effect of the amino acid identity on a specific property and also how such behaviour is highly influential on the self-assembly and supramolecular structure.

Finally, the metallocarbonate core exhibited in the trinuclear Ni^II^ com­plex proved to be rather unique since, to our knowledge, there is no information in the literature of other Ni^II^ systems showing this exact structure. Magnetic susceptibility and magnetization data support weak ferromagnetic exchange interactions within this novel symmetrical Ni_3_–μ_3_-CO_3_ core.

## Supplementary Material

Crystal structure: contains datablock(s) 1ni-sc, 2ni-sc. DOI: 10.1107/S2052520620010008/lo5080sup1.cif

Structure factors: contains datablock(s) 1ni-sc. DOI: 10.1107/S2052520620010008/lo50801ni-scsup2.hkl

Structure factors: contains datablock(s) 2ni-sc. DOI: 10.1107/S2052520620010008/lo50802ni-scsup3.hkl

CSD statistical analysis; additional information regarding synthesis and characterization (IR spectra, thermal studies, microscopy images); XRD studies: single crystal XRD relevant tables, additional figures, powder XRD data and other additional structural analysis; supplementary magnetic measurements. DOI: 10.1107/S2052520620010008/lo5080sup4.pdf

CCDC references: 1984177, 1984178

## Figures and Tables

**Figure 6:**
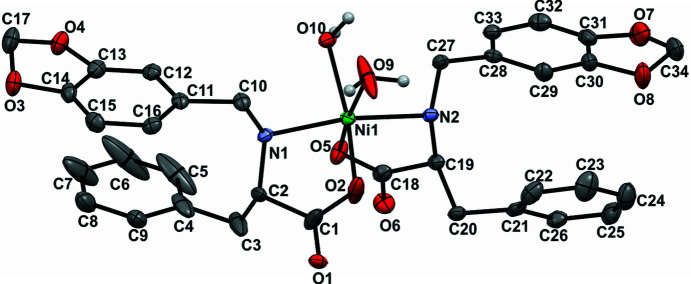
The structure of com­plex **2Ni** determined by single-crystal X-ray diffraction (**2Ni-SC**), viewed along the *bc* plane. Some H atoms, water and DMF solvent molecules, and disordered moieties have been omitted for clarity. Displacement ellipsoids are drawn at the 50% probability level. Colour code: O atoms red, C gray, N violet–blue and Ni green.
