# Supplementary material for: Nickel(II) com­plexes based on l-amino-acid-derived ligands: synthesis, characterization and study of the role of the supramolecular structure in carbon dioxide capture
Source: Acta Crystallogr B Struct Sci Cryst Eng Mater. 2020 Sep 3;76(Pt 5):825–38. doi: 10.1107/S2052520620010008 (PMC7535065; doi:10.1107/S2052520620010008)
Supplement: Supplementary file 4 [file b-76-00825-sup4.pdf]

STRUCTURAL SCIENCE
CRYSTAL ENGINEERING
MATERIALS

Volume 76 (2020)

Supporting information for article:

Nickel(II) complexes based on L-amino acids derived ligands: synthesis, characterization and study of the role of the supramolecular structure in the carbon dioxide capture

Andrea Rivas Marquina, Federico Movilla, Olga Carolina Sánchez Montilva, Eva Rentschler, Luca Carrella, Pablo Albores and Florencia Di Salvo

Supporting Information

Nickel(II) complexes based on L-amino acids derived ligands: synthesis, characterization and study of the role of the supramolecular structure in the carbon dioxide capture

Andrea Rivas Marquina,[a] Federico Movilla,[a] Olga Sánchez Montilva,[a] Eva Rentschler,[b] Luca Carrella,[b] Pablo Albores,[a] and Florencia Di Salvo.*,[a]

[a] Departamento de Química Inorgánica, Analítica, y Química Física, Facultad de Ciencias Exactas y Naturales, Universidad de Buenos Aires/INQUIMAE-CONICET, Intendente Güiraldes 2160, Pabellón 2, Piso 3, Ciudad Universitaria, C1428EGA Ciudad de Buenos Aires, Argentina; E-mail: flor@qi.fcen.uba.ar

[b] Johannes Gutenberg University Mainz Chemistry, Institute of Inorganic and Analytical Chemistry, Duesbergweg 10-12, 55128 Mainz, Germany.

Supporting Information Index

1. Analysis of carbonate-metal complexes using the Crystallographic Data Base (CSD) **2**

2. Synthesis and characterization **7**

IR and UV-Vis Spectra 11

Thermal studies 16

3. XRD studies **19**

Single Crystal XRD tables 19

Powder XRD studies 30

Additional structural analysis 31

4. Supplementary Magnetic Measurements **38**

5. Supplementary Information References **40**

1. Analysis of carbonate-metal complexes using the Crystallographic Data Base (CSD)

References S1-S15 show all L-Tyr-based-ligands Ni(II) coordination complexes included at the CSD, respectivelly.S16 References S6-S15 correspond to unsubstituted L-Tyr (10 hits) and S17-S20 to L-Phe (8 hits) Ni(II)-systems.

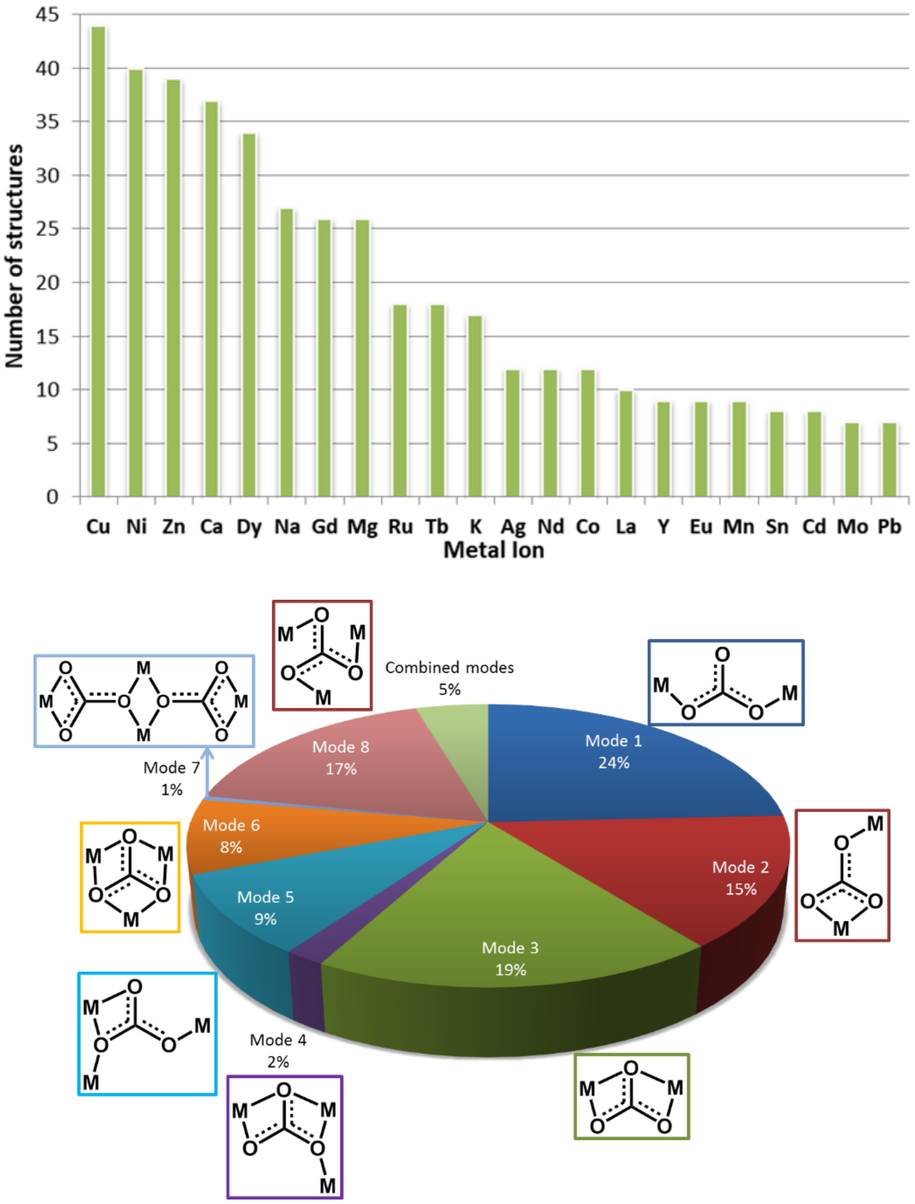

Figure S1 Top: number of structures of metallocarbonate complexes in the CSD including all coordination modes, sorted by metal. For clarity, results include metal ions with more than five structures reported. Bottom: Distribution of carbonate coordination modes for all metallocarbonate complexes reported in the CSD.

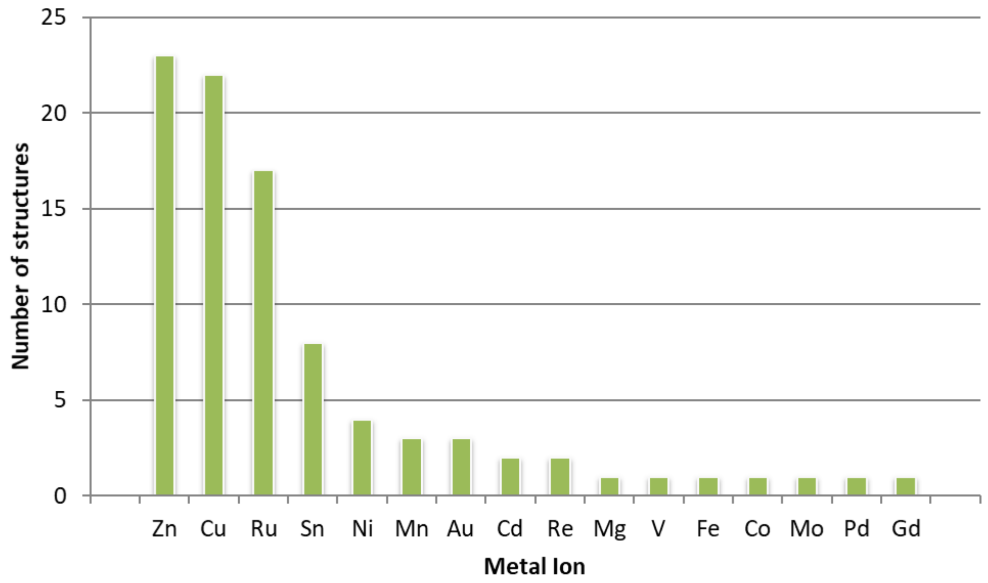

Figure S2 Number of structures of metallocarbonate complexes in the CSD bearing only coordination mode 8 for the carbonate, sorted by metal.; for Ni(II) 4 hits are found.

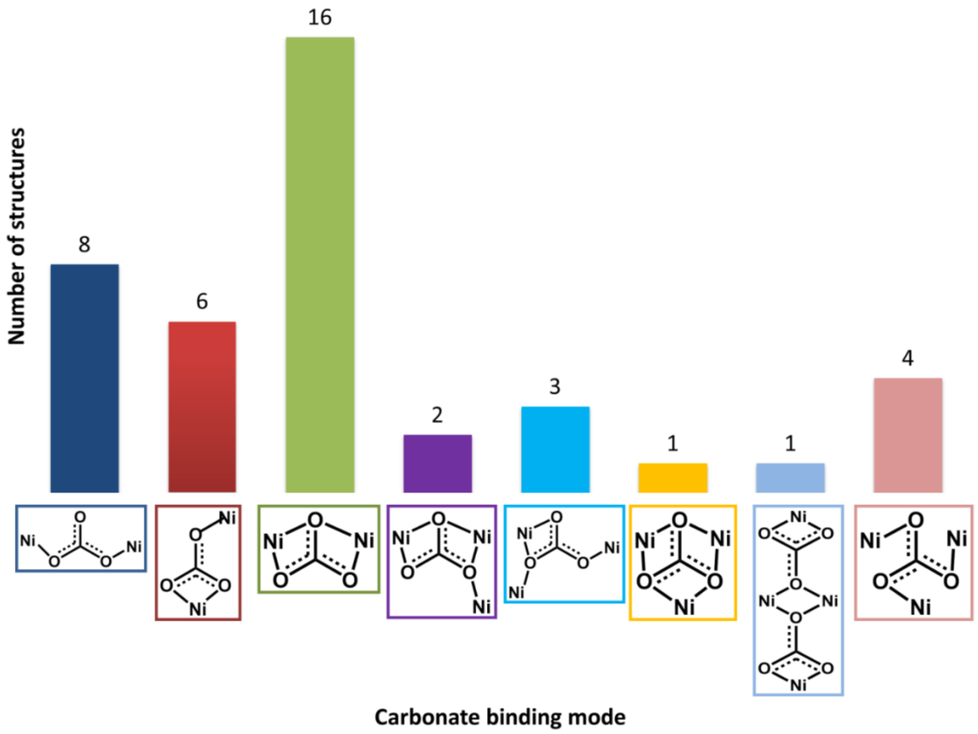

Figure S3 Number of structures of Ni(II)-carbonate complexes found at the CSD, sorted by carbonate binding mode; 4 structures are found for mode 8.

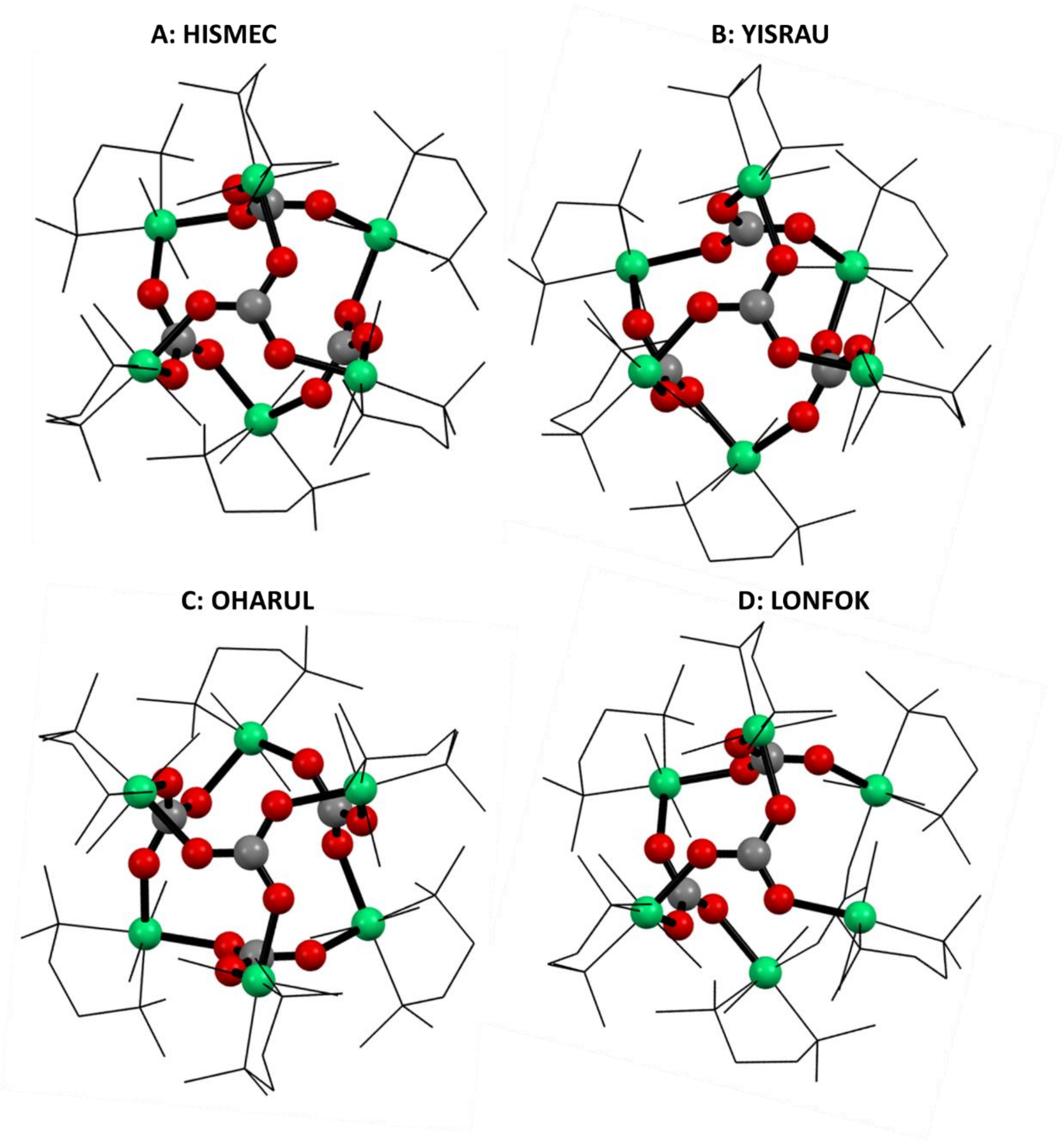

Figure S4 Structures of the four Ni-carbonate systems found in the CSD exhibiting bridging mode 8 (the same found in **1Ni-SC**); ID code (A) **HISMEC** (B) **YISRAU** (C)[S21] **OHARUL** (D)[S22] **LONFOK** structures.[S23] Hydrogen atoms are not shown for clarity. Ni-carbonate cores are shown in ball&sticks, rest of the molecules in wireframes.

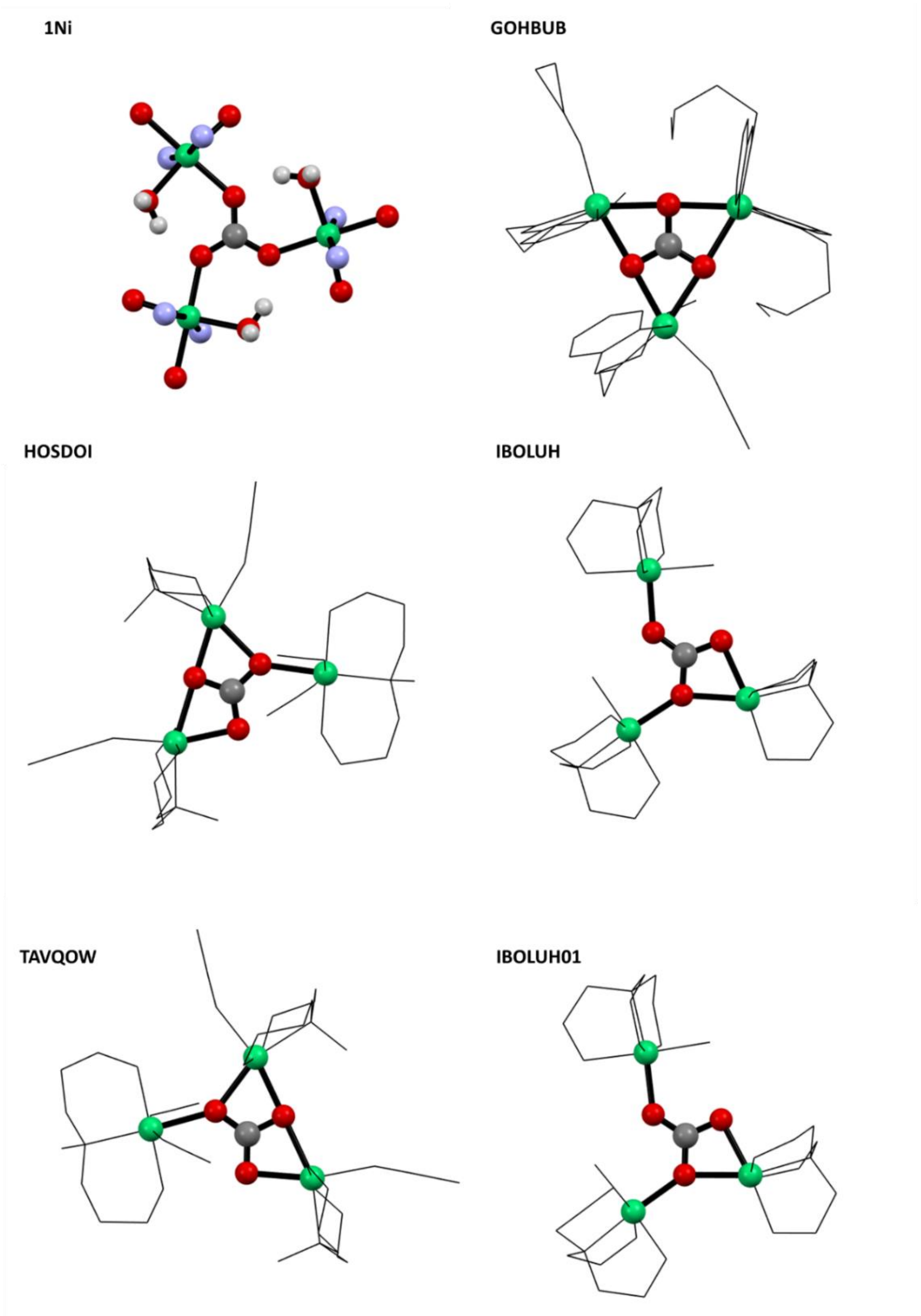

Figure S5 **1Ni** and the 5 structures of Ni_3-carbonate complexes found in the CSD with ID codes (A) **GOHBUB** (B) **HOSDOI** (C) **IBOLUH** (D) **IBOLUH01** (E) **TAVQOW**.[S24-S28] Hydrogen atoms are not shown for clarity. Ni-carbonate cores are shown in ball&sticks, rest of the molecules in wireframes. Systems with only one Ni center but different coordination modes for the carbonate bridge

In the structures with ID codes HISMEC, YISRAU and in one of the two units of LONFOK additional H-bonds are displayed for each O atom of the carbonates, having an average distances of 1.766, 1.773 and 1.724 Å, respectively, slightly shorter that the value for **1Ni-SC** of 1.837 Å (Figure 7 of the MS). For the structure with ID code OHARUL, since the H atoms of the coordinated water molecules were not refined, it was not analyzed.

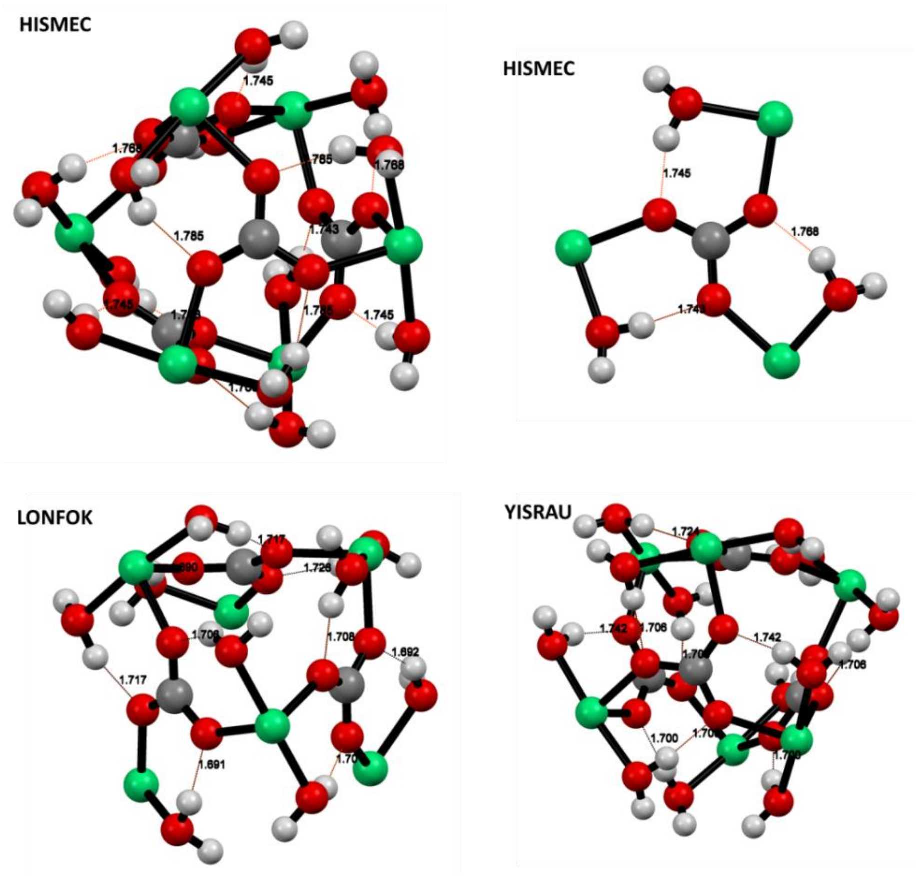

Figure S6 H-bond sets between the coordinated water molecules and the carbonate bridging ligand for structures found at the CSD related to **1Ni-SC**.[S21-S23]

2. Synthesis and characterization

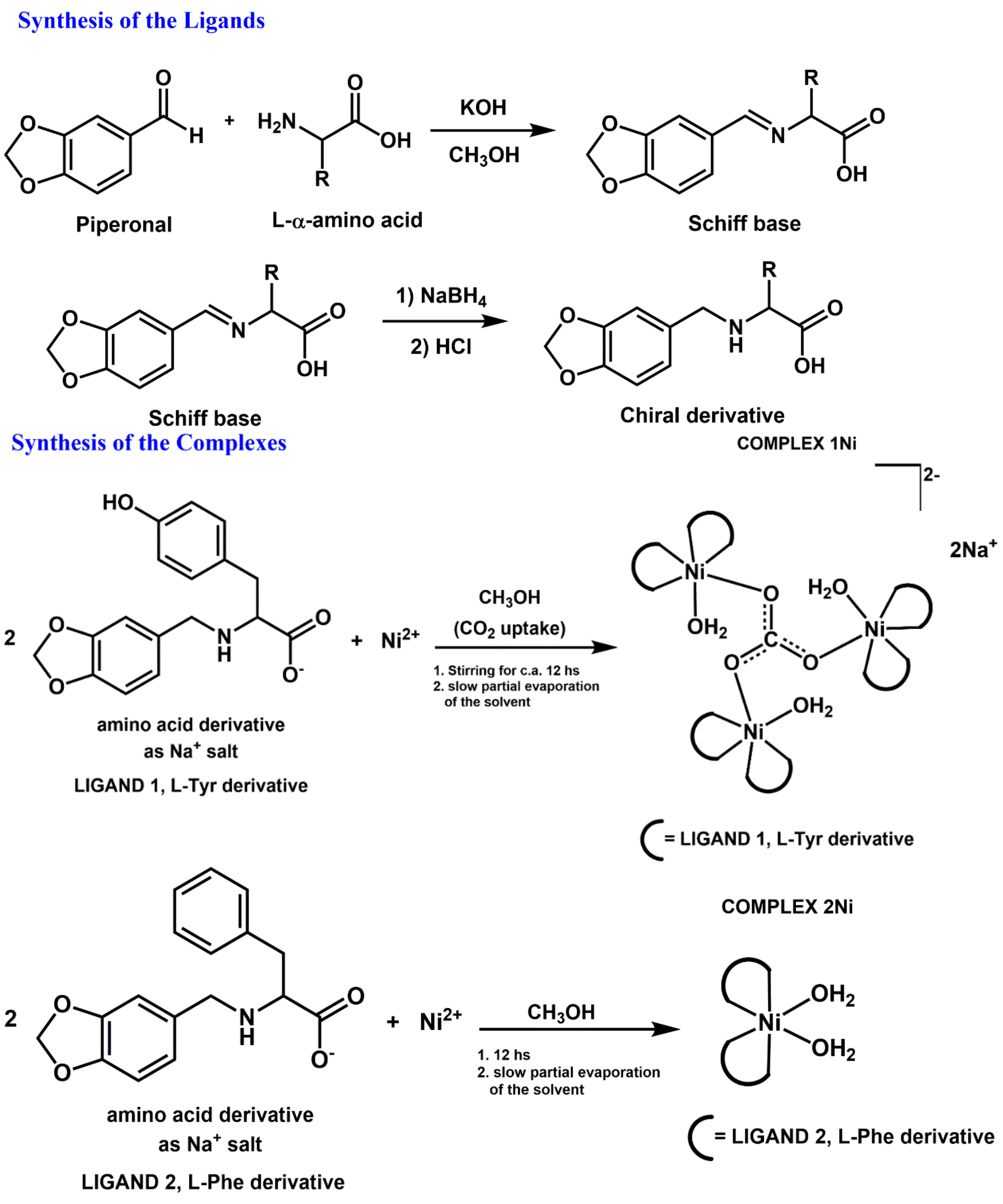

Figure S7 Top: Reaction procedure for the obtaining of ligands 1 and 2, R = p-phenol for L-Tyr and phenyl for L-Phe derivative, respectively. Bottom: synthesis of the complexes **1Ni** and **2Ni**

LIGAND 3

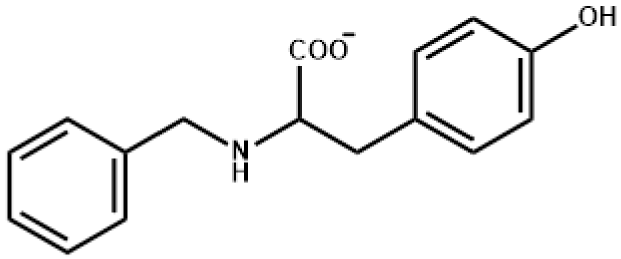

Figure S8 Scheme of Ligand **3**, derivative obtained using L-tyr and benzaldehyde.

Synthesis of Ligand 3. It was prepared following the procedure described for **1** except that 200mg (1.1mmol) of L-Tyr and 88mg de NaOH (2.2mmol) were used, and after the addition of $NaBH_4$ the reaction mixture was stirred at RT and not under reflux. Yield: 150mg (50.2%). ^{1}H NMR (D_2O/NaOD): δ 2.71 (d, 2H, *J* = 6Hz), 3.22 (t, 1H, *J* = 6Hz), 3.51 (d, 1H, *J* = 12Hz), 3.70 (d, 1H, *J* = 12Hz), 6.50 (d, 2H, *J* = 8Hz), 6.90 (d, 2H, *J* = 8Hz), 7.25 – 7.35 (m, 5H). ^{13}C NMR (D_2O/NaOD): δ 38.11, 50.98, 64.76, 118.52, 123.51, 127.27, 128.58, 128.61, 130.49, 138.96, 164.50, 181.69. Selected FTIR peaks (KBr, cm^{-1}): 3174(s), 3100 - 2400(w), 1607(s), 1582(s), 1516(s), 1437(m), 1397(s), 1259(s), 829(w), 749(w), 699(w). Selected UV-visible bands (in nm H_2O/NaOH solution): λ: 295nm.

Synthesis of complex 3Ni starting form $Ni(NO_3)_2{\cdot}6H_2O$. In a 5mL vial, 6mg (0.02mmol) of $Ni(NO_3)_2{\cdot}6H_2O$ were dissolved in 1mL of methanol and then, a solution of the sodium salt of ligand **3** was added. The solution of the ligand was prepared using 5mg (0.018mmol) of **3**, 1.6mg (0.04mmol) of NaOH and 1mL of methanol. The resulting blue-green solution of the complex was stirred overnight and then after removing the cap of the vial and covering with parafilm foil with small holes, it was open to the air and left for slow evaporation of the solvent at room temperature. After c.a. 3 days, light-blue crystalline aggregates were obtained. A lot of different crystallization protocols were explored but unfortunately, so far any of them were successful, and thus we were not able to obtain suitable crystalline material to perform single crystal x-ray experiments. Selected FTIR peaks (KBr, cm^{-1}): ν(O-H) = 3562 (s), ν(N-H) = 3256 (s), ν(C=O) = 1610 (s), ν(C=C) = 1585 (s), ν(C-O) = 1514, 1442 (m), ν(C-H) = 1395 (s), ν(C-O) = 1263 (m), ν(C-O) = 1071 (m).

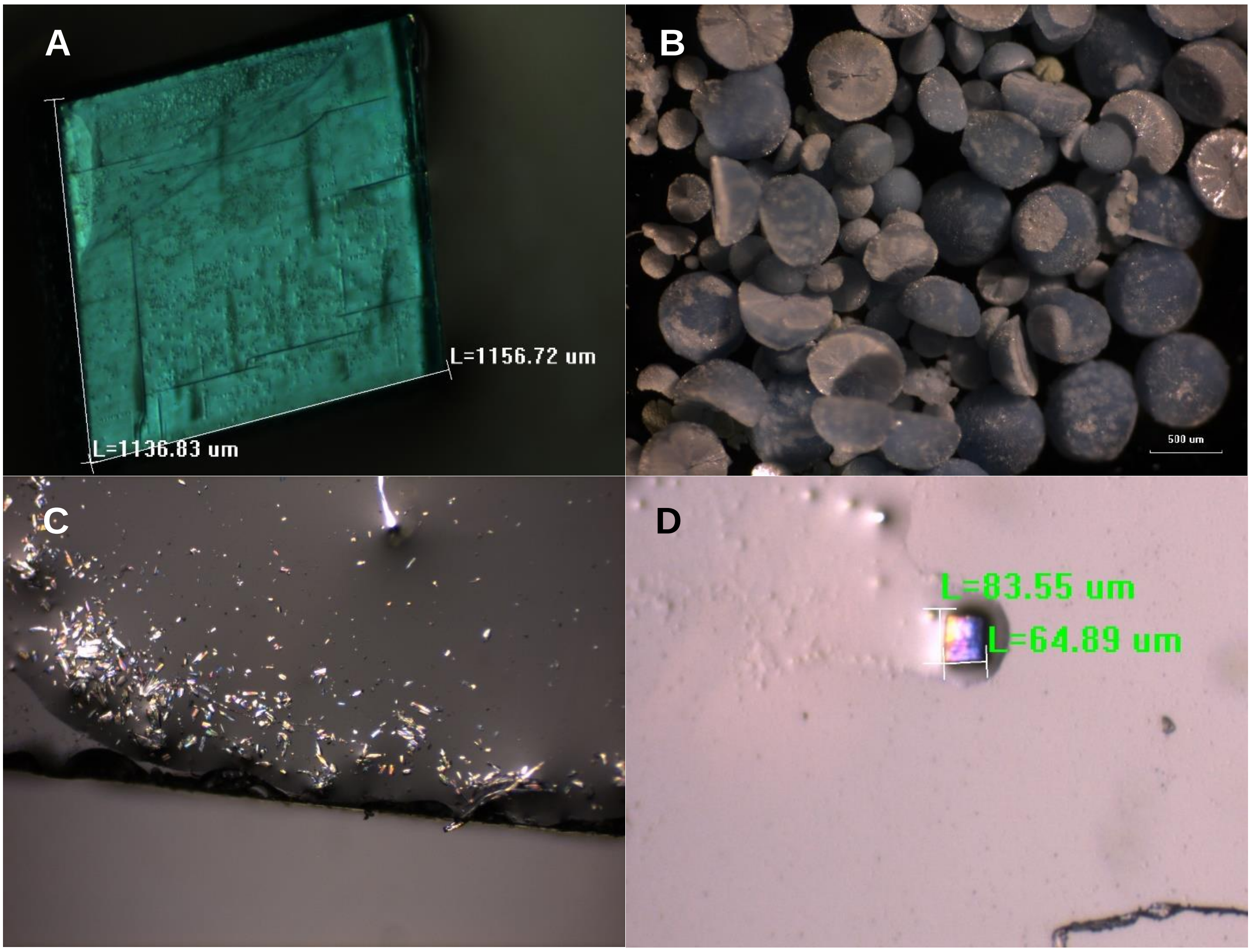

Figure S9 Images taken using Polarized Light Microscopy; A: **1Ni-SC**, B: **2Ni-CA**, C&D: **2Ni-SC**

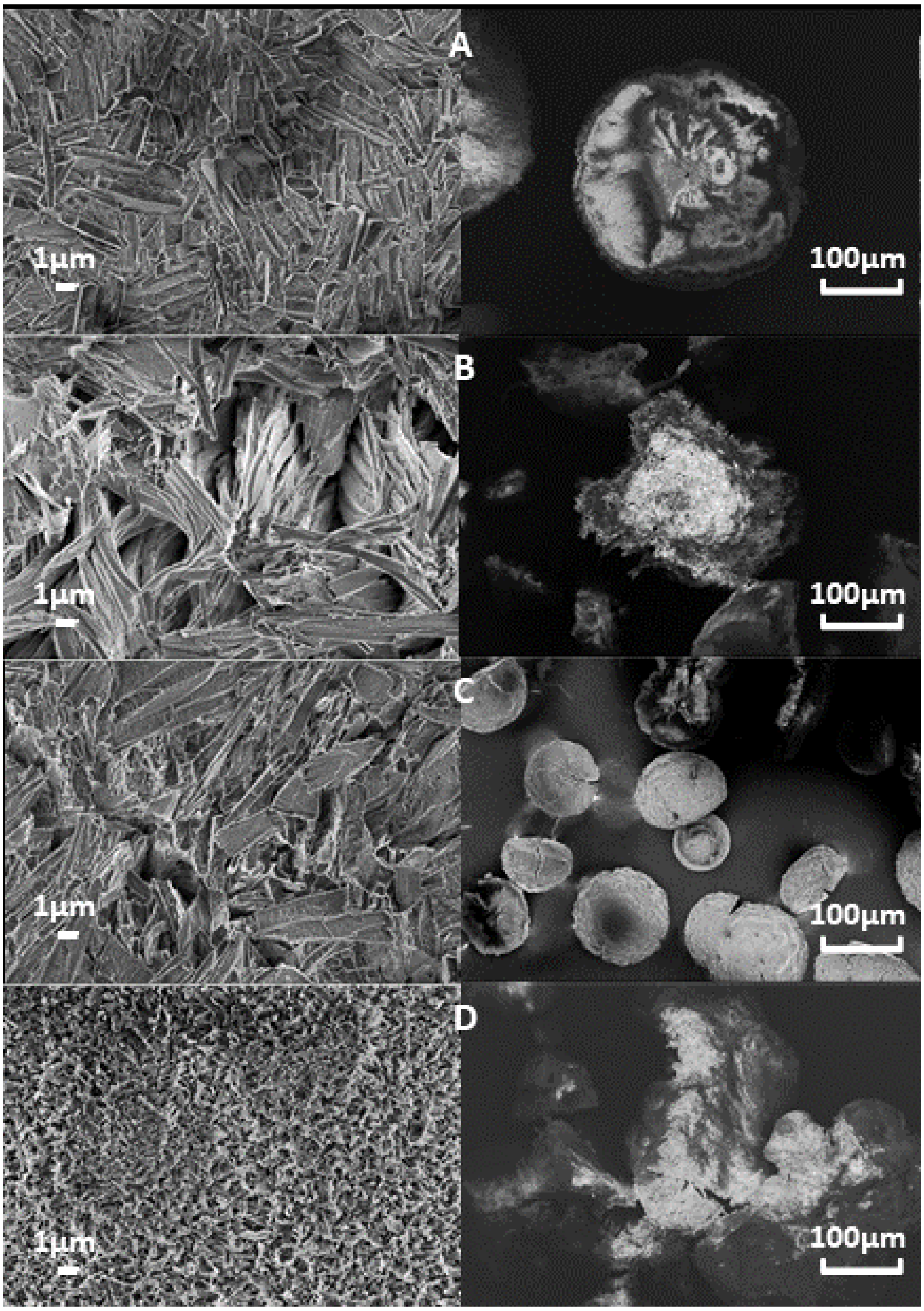

Figure S10 SEM images of **2Ni-CA** starting from different Ni(II) salts. A: $Ni(NO_3)_2 \cdot 6H_2O$, B: $NiAc_2 \cdot 4H_2O$, C: $NiSO_4 \cdot 6H_2O$, D: $NiCl_2 \cdot 6H_2O$.

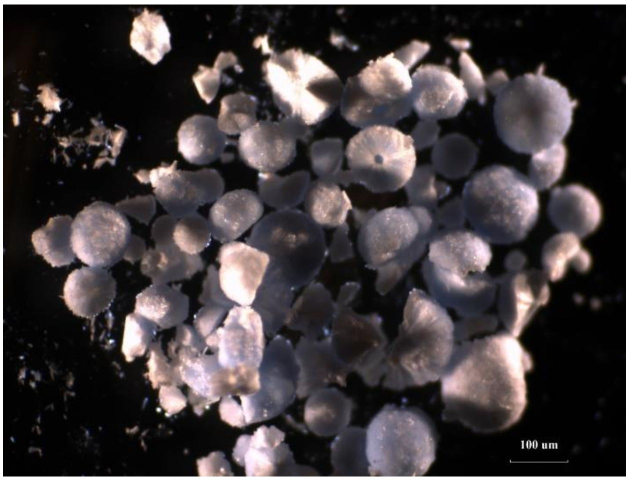

Figure S11 Images of **3Ni** using Polarized Light Microscopy

IR and UV-Vis Spectra

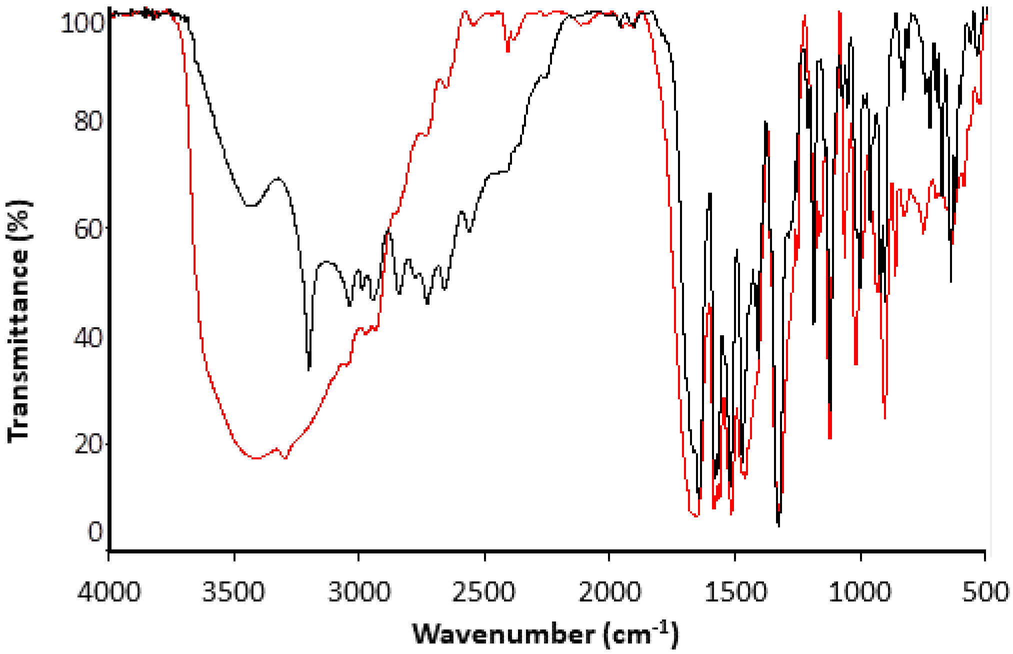

Figure S12 FT-IR spectra (KBr pellet) of ligand **1** (black line) and complex **1Ni** (red line).

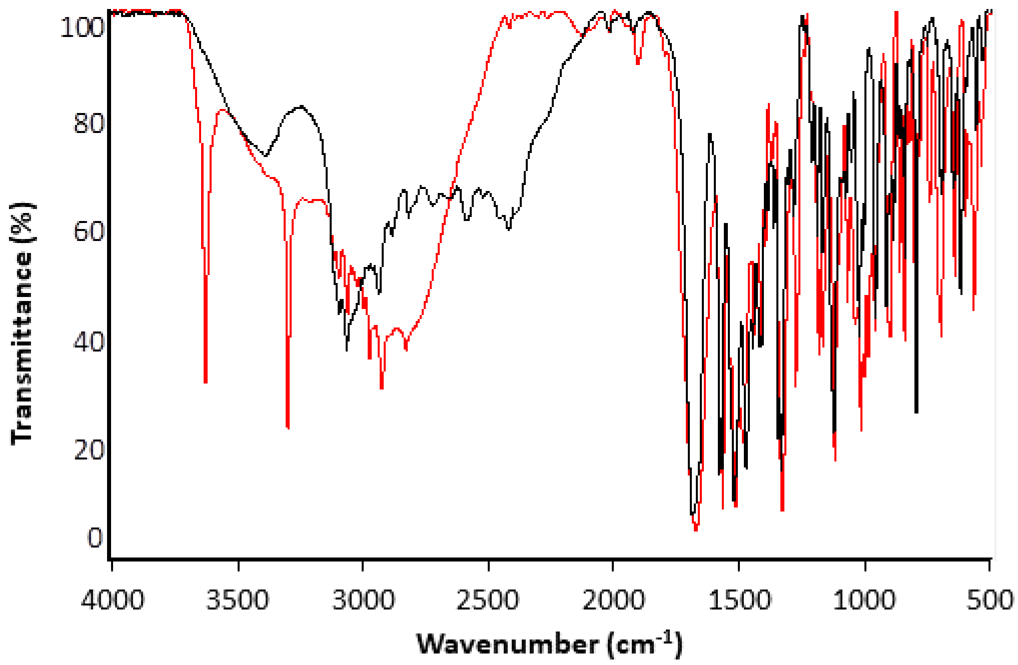

Figure S13 FT-IR spectra (KBr pellet) of ligand **2** (black line) and complex **2Ni** (red line) in KBr pellet.

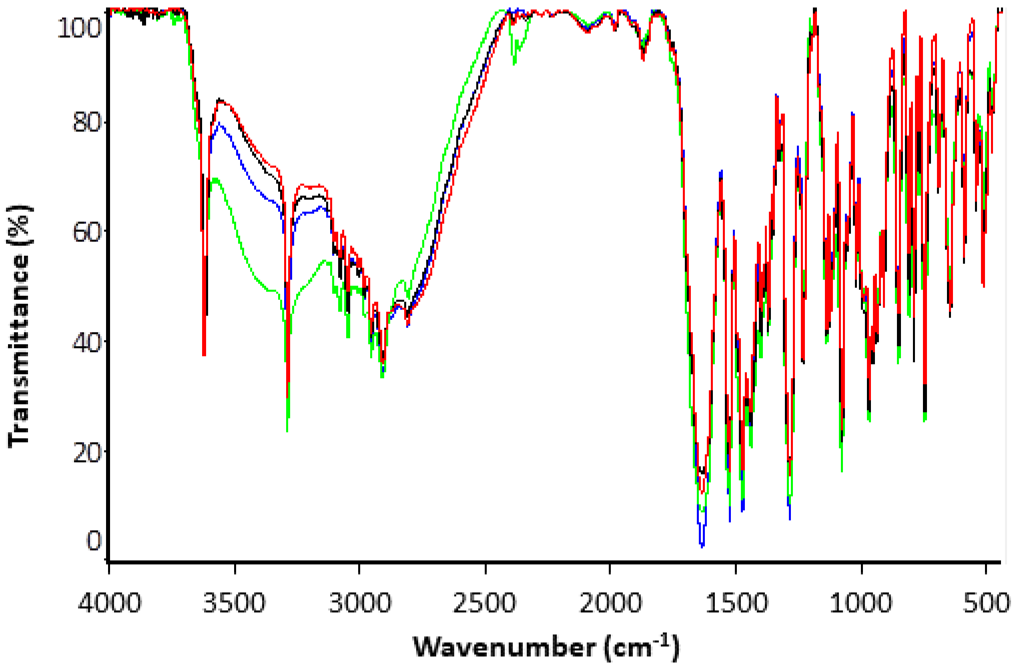

Figure S14 FT-IR spectra of complex **2Ni** starting using different Ni(II) salts. $NiCl_2·6H_2O$ (black line), $NiAc_2·4H_2O$ (blue line), $NiSO_4·6H_2O$ (green line) and $Ni(NO_3)_2·6H_2O$ (red line).

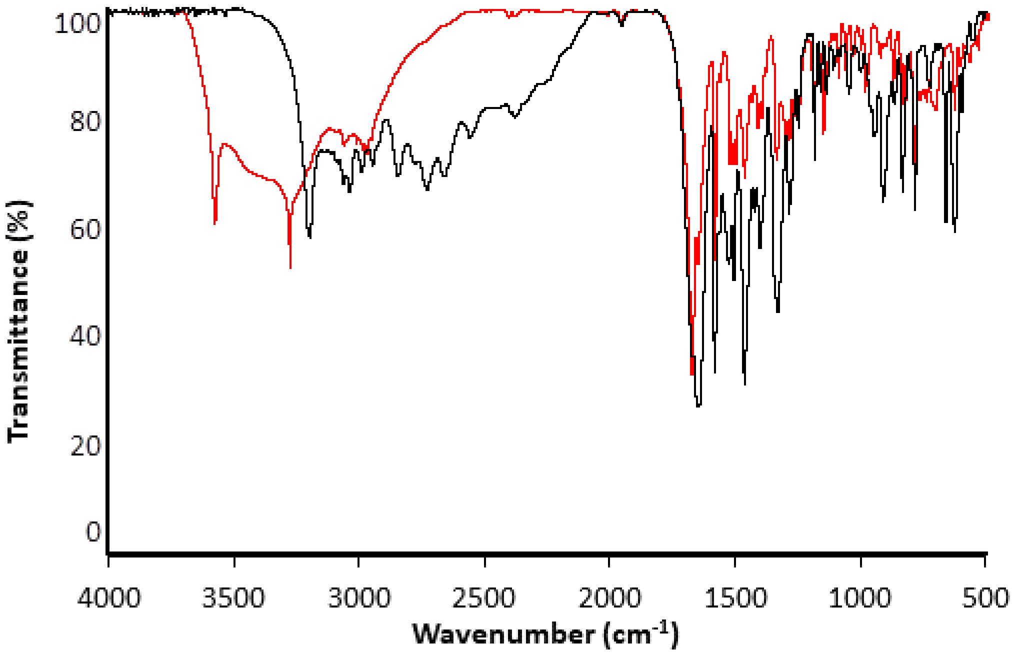

Figure S15 FT-IR spectra (KBr pellet) of ligand **3** (black line) and complex **3Ni** (red line).

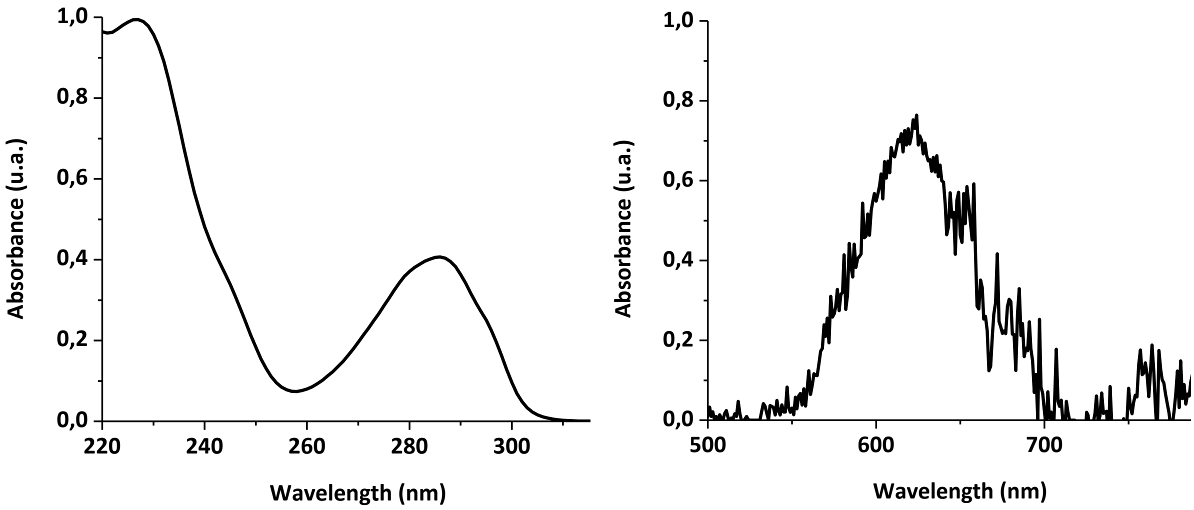

Figure S16 Normalized UV spectra (left) and UV-Vis spectra (right) of **1Ni** in methanol.

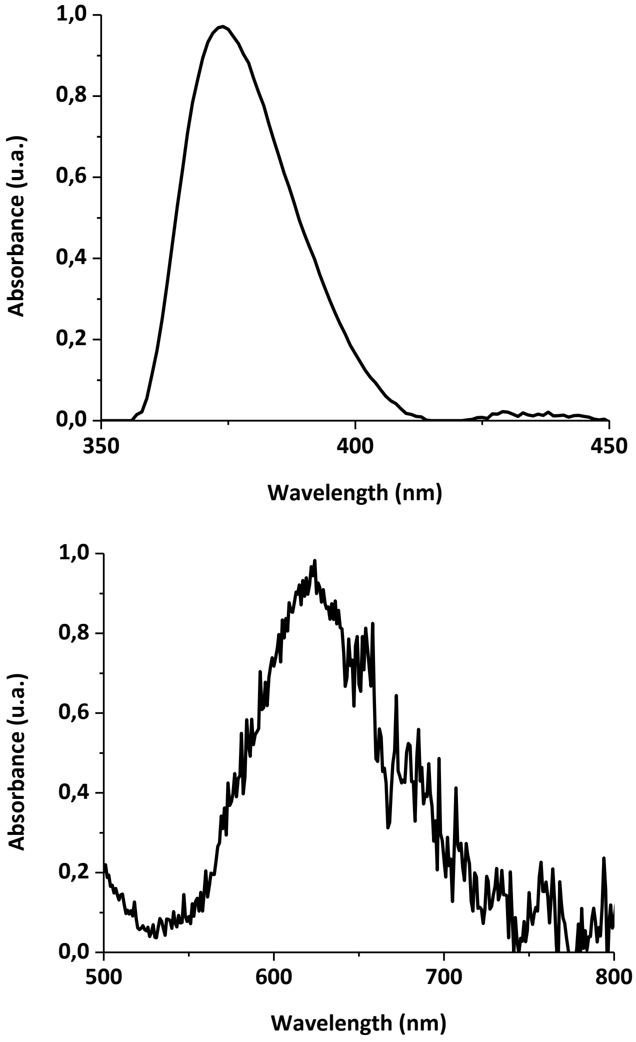

Figure S17 Normalized UV spectra (left) and UV-Vis spectra (right) of **2Ni** in DMSO.

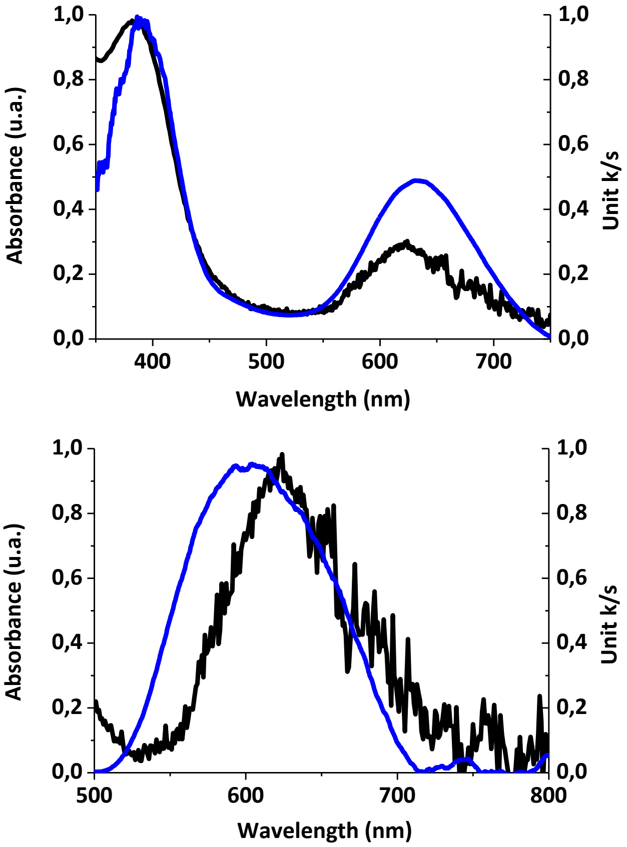

Figure S18 Normalized UV-Vis spectra (black line) and Kubelka-Munk transformed reflectance spectra (blue line) of **1Ni** (left) and **2Ni** (right).

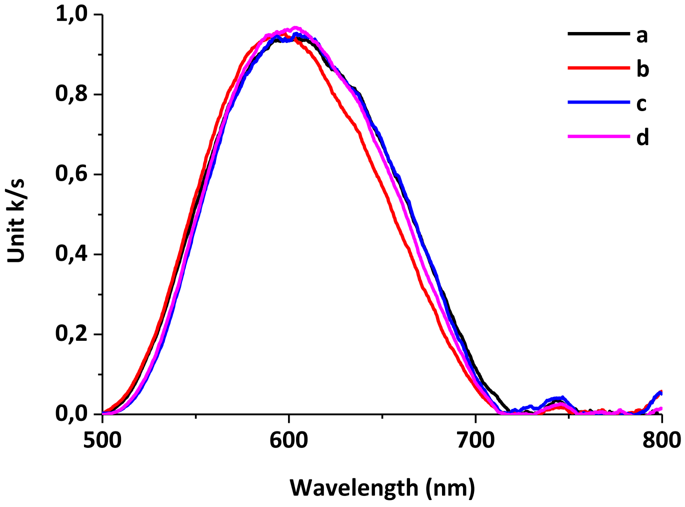

Figure S19 Normalized Kubelka-Munk transformed reflectance spectra of **2Ni** starting from different Ni(II) salts. a) $NiAc_2 \cdot 4H_2O$, b) $NiCl_2 \cdot 6H_2O$, c) $Ni(NO_3)_2 \cdot 6H_2O$, d) $NiSO_4 \cdot 6H_2O$.

Thermal studies

In order to quantify the amount of solvent in the structure present in the voids and inform a more accurate molecular formula, Thermo Gravimetric Analyses (TGA), Differential Scanning Calorimetric studies (DSC) and CHNS Elemental Analysis were performed on fresh crystals of **1Ni-SC** removing carefully all the remaining supernatant. The results obtained with these methodologies agreed and confirmed that in the structure there are 11 molecules of H_2O and 16 of MeOH per unit of the trimeric complex. Regarding polycrystalline material (**1Ni-PM**) and amorphous solids (**1Ni-CA**) obtained after using different drying methods, TGA, DSC and elemental analysis were also performed. In such cases the results were used to monitor the loss of solvent and estimate a molecular weight for other experiments, such as magnetic behavior studies.

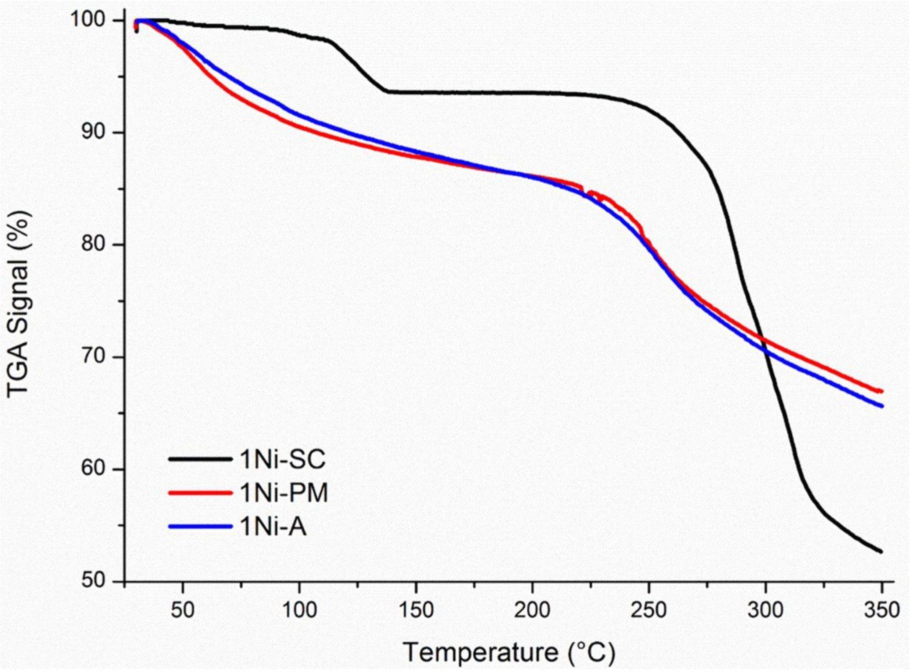

Figure S20 Thermogravimetric analysis obtained for **1Ni** single crystals (**SC**), polycrystalline material (**PM**) and amorphous solid (**A**). The loss of considerable amount of solvent is observed for **1Ni-SC**. Smaller amount of solvent is observed for both polycrystalline and amorphous material.

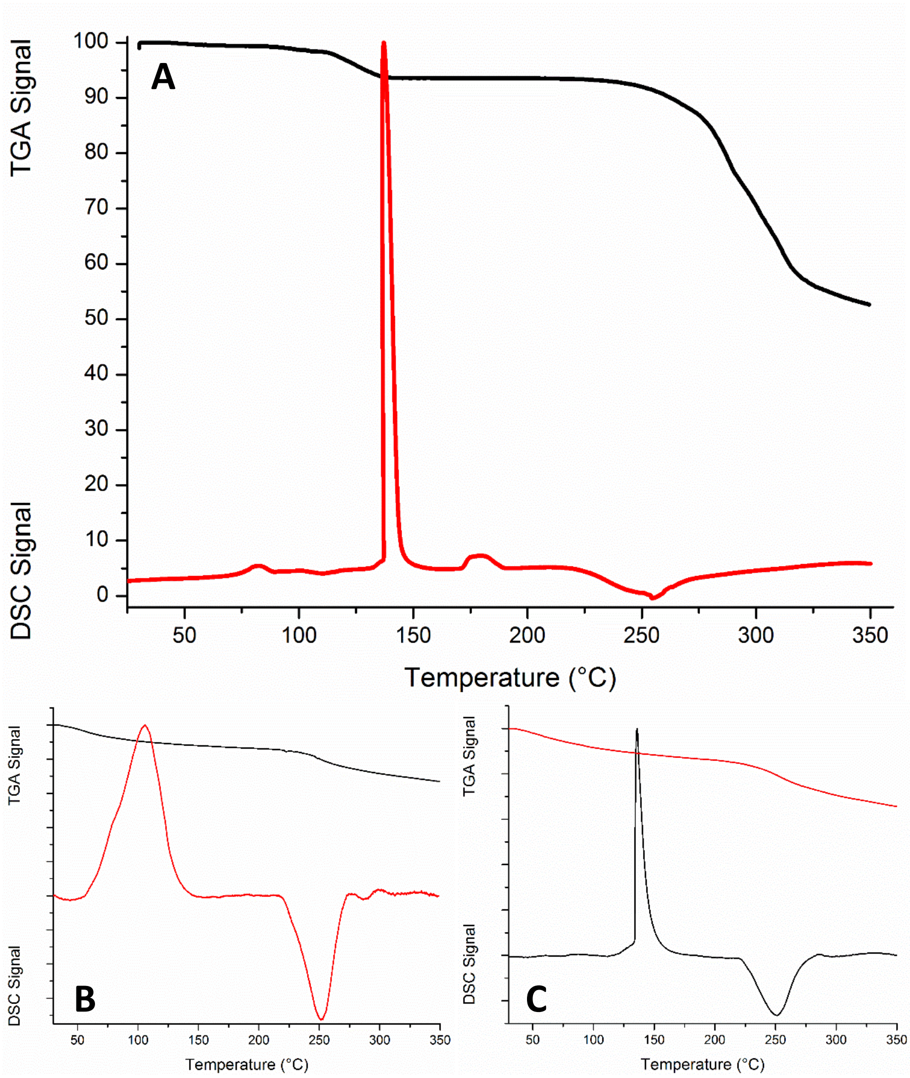

Figure S21TGA and DSC analysis superposed for **1Ni-SC** (A) **1Ni-PM** (B) and **1Ni-A** (C). In all of them, thermograms show an intense exothermic peak in the region where the solvent molecules are lost. After that, a smaller endothermic peak is observed, which it is associated with structural-solvent loss (coordinated water molecules). Finally, the decomposition of the complexes is observed.

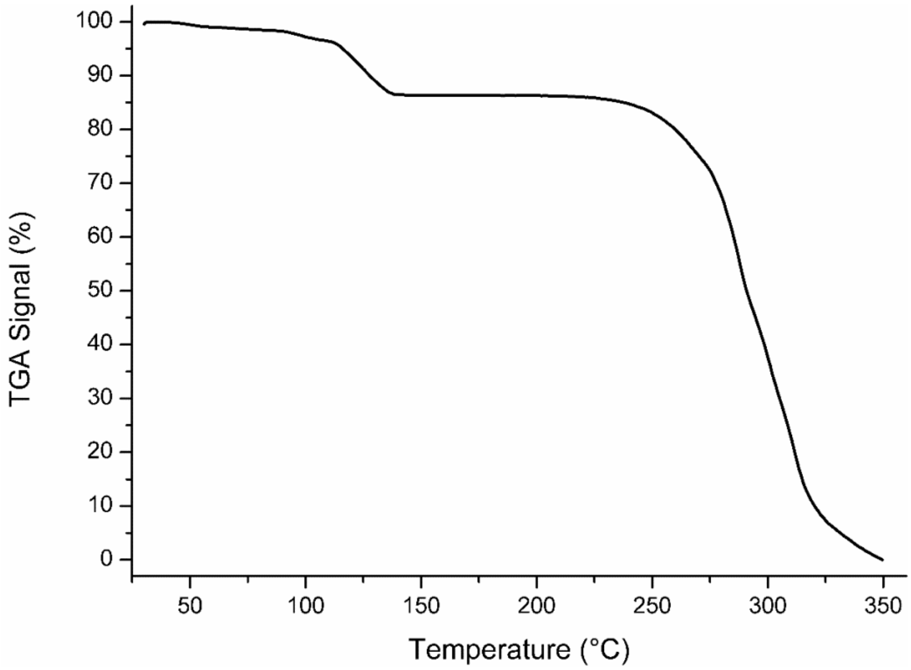

Figure S22 TGA obtained for **2Ni-CA**. A moderate mass loss at the beginning, associated with a residual superficial solvent lost, could be appreciated. After that, a strong mass loss due to the decomposition of the complex is observed.

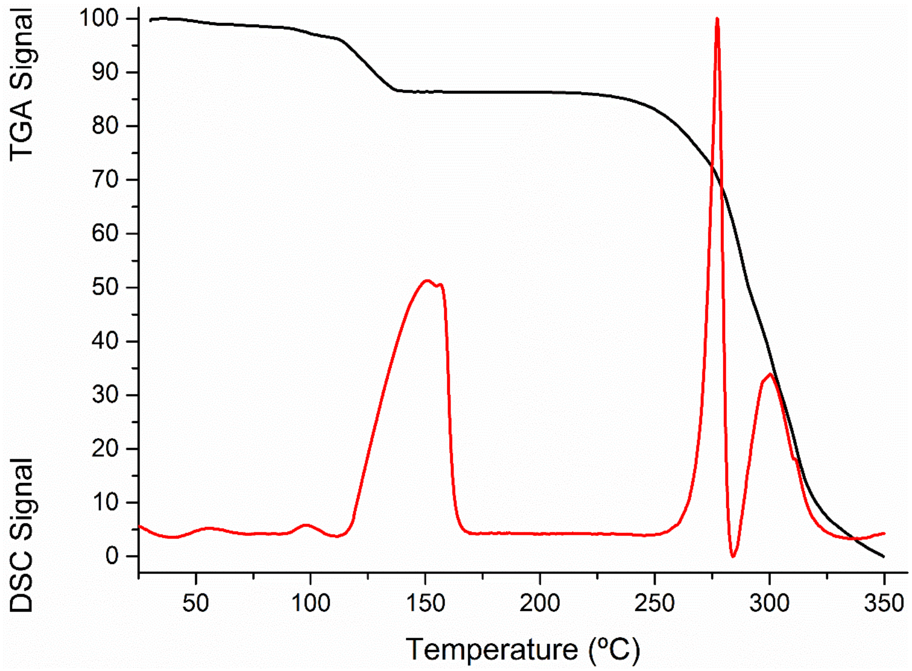

Figure S23 TGA and DSC analysis superposed for **2Ni-CA**. An intense exothermic peak is seen in the region where the solvent molecules are lost. After that, another highly exothermic peak is observed, which it is associated with structural-solvent loss (coordinated water molecules). Finally, the decomposition of the complex appears as a moderate peak at the end.

3. XRD studies

Single Crystal XRD tables

Crystals of **1Ni-SC** suitable for XRD studies were obtained after slow evaporation of the reaction solvent. After removing the crystals from their mother liquor, they were immediately covered by mounting oil and after a fast inspection, a suitable crystal was mounted in a loop and placed in the diffractometer at low temperature. Several crystals were tested using an Oxford Diffraction Gemini E lab diffractometer with Mo Kα (λ = 0.71 Å) radiation, but due to the partial loss of solvent molecules during the experiment and the decrease of the crystal quality, the resulting data were not good enough to give place to acceptable resolutions. The full data collection was planned using the CrysAlis Pro strategy tool[S29] and data were reduced using CrysAlis Pro programme. A Gaussian method implemented in WinGX[S30] or a numerical model[S31] was used for the absorption correction. Crystals of complex **1Ni** obtained from different Ni(II) salts were studied by single crystal XRD using an Oxford Diffraction Gemini E lab diffractometer with Mo Kα (λ = 0.71 Å) radiation as well. Full data collection for **1Ni-SC** and **2Ni-SC** were performed at a Synchrotron beamline.

Table S1. Crystal data and structure refinement for **1Ni-SC** and **2Ni-SC**.

	**1Ni-SC**	**2Ni-SC**
**Formula**	$C_{103}H_{102}N_6Na_2Ni_3O_{36}$	$C_{34}H_{40}N_2NiO_{10}.2H_2O.C_3H_7NO$
**ρ_{calc} (g cm^{-3})**	1.024	1.439
**μ (mm^{-1})**	0.627	0.893
**Mol. Weight**	2222.03	800.48
**Colour**	Green	Light green-blue
**Size (mm^3)**	1.00 × 0.50 × 0.50	0.10 × 0.10 × 0.05
**T (K)**	100.1(1)	100.0(1)
**Crystal System**	trigonal	orthorhombic
**Flack**	0.035(3)	0.022(2)
**Hooft**	0.0518(7)	0.0203(13)
**Space Group**	*R*3	$P2_12_12$
**a (Å)**	20.020(2)	14.910(3)
**b (Å)**	20.020(2)	33.6200(9)

**c (Å)**	31.130(10)	7.370(2)
**α (º)**	90	90
**β (º)**	90	90
**γ (º)**	120	90
**V ($Å^3$)**	10805(4)	3694.4(12)
**Z**	3	4
**Z'**	1/3	1
**Wavelength (Å)**	0.79983	0.82610
**Radiation type**	Synchrotron	Synchrotron
**Diffractometer**	Custom air-bearing with MK3 mini-kappa	Custom air-bearing with MK3 mini-kappa
**Detector**	Pilatus2M	Pilatus2M
**Monochromator**	Si(111) double-crystal	Si(111) double-crystal
**Absorption correction**	Empirical absorption correction using XDS	Empirical absorption correction using XDS
**$θ_{min}$ (º)**	1.979	1.408
**$θ_{max}$ (º)**	29.997	28.500
**Measured Refl.**	287004	113805
**Independent Refl.**	9751	5935
**Refl. with I > 2(I)**	9583	5905
**R_{int}**	0.0297	0.0383
**Parameters**	466	520
**Restraints**	1	0
**Largest Peak**	1.043	0.563
**Deepest Hole**	–0.468	-0.540
**Goodness-of-fit on F^2**	1.111	1.047
**wR_2 (all data)**	0.1859	0.0786

**wR_2**	0.1842	0.0788
**R_1 (all data)**	0.0715	0.0343
**R_1**	0.0710	0.0341

Table S2. Cell parameters for **1Ni-SC** obtained using different Ni(II) salts

Salt	Space Group	a/Å	b/Å	c/Å	α/°	β/°	γ/°	V/Å^3	T / K
$NiAc_2$	*R*3	20.02	20.23	30.75	90.51	89.6	120.2	10766	298
$NiSO_4$	*R*3	19.96	19.91	30.71	90.15	90.03	119.64	10607	298
$NiAc_2$	*R*3	20.27	20.29	30.83	91.3	89.7	120.1	10973	298
$NiCl_2$	*R*3	19.67	19.67	30.74	90	90	120	10300	100

Table S3. Bond Lengths in Å for **1Ni-SC.** **Table S4.** Bond Angles in (º) for **1Ni-SC.**

Atom	Atom	Length (Å)	Atom	Atom	Atom	Angle (º)
Ni1	O2	2.057(3)	O2	Ni1	O4	89.97(13)
Ni1	O3	2.049(4)	O2	Ni1	O5	172.12(13)
Ni1	O4	2.087(3)	O2	Ni1	N6	91.78(13)
Ni1	O5	2.061(3)	O2	Ni1	N7	100.07(15)
Ni1	N6	2.113(4)	O3	Ni1	O2	84.63(13)
Ni1	N7	2.106(4)	O3	Ni1	O4	172.62(14)
O2	C8	1.288(3)	O3	Ni1	O5	97.42(15)
O21	C13	1.260(6)	O3	Ni1	N6	89.73(15)
O3	C9	1.258(7)	O3	Ni1	N7	82.13(16)
O4	Na12	2.676(7)	O4	Ni1	N6	95.48(14)
O5	C13	1.242(6)	O4	Ni1	N7	93.90(16)
O78	C68	1.368(8)	O5	Ni1	O4	88.61(14)

N6	C15	1.477(6)	O5	Ni1	N6	80.64(14)
N6	C16	1.489(6)	O5	Ni1	N7	87.76(15)
O58	C45	1.387(7)	N7	Ni1	N6	164.88(15)
O58	C71	1.412(8)	C8	O2	Ni1	132.6(2)
O81	C73	1.391(10)	C9	O3	Ni1	114.7(4)
O20	C9	1.272(7)	Ni1	O4	Na12	105.6(3)
O20	Na32	2.606(12)	C13	O5	Ni1	116.2(3)
N7	C18	1.484(7)	C15	N6	Ni1	108.4(3)
N7	C19	1.482(7)	C15	N6	C16	113.8(4)
O72	C71	1.416(11)	C16	N6	Ni1	110.7(3)
O72	C59	1.388(8)	C45	O58	C71	105.6(5)
C15	C13	1.534(7)	C9	O20	Na32	125.2(5)
C15	C23	1.552(8)	C18	N7	Ni1	108.2(3)
O76	C65	1.368(12)	C19	N7	Ni1	115.2(3)
O76	C77	1.34(3)	C19	N7	C18	112.7(4)
O67	C53	1.406(14)	C59	O72	C71	106.1(5)
O67	C77	1.47(2)	N6	C15	C13	111.0(4)
C42	C86	1.394(8)	N6	C15	C23	111.0(4)
C42	C55	1.384(9)	C13	C15	C23	106.7(4)
C35	C24	1.411(7)	O21	C13	C15	117.1(5)
C35	C45	1.380(7)	O5	C13	O21	124.0(5)
C86	C23	1.506(7)	O5	C13	C15	118.8(4)
C86	C43	1.391(10)	C77	O76	C65	106.2(16)
C24	C16	1.519(6)	C53	O67	C77	100.9(13)
C24	C36	1.390(8)	C55	C42	C86	120.8(7)
C73	C62	1.368(11)	C45	C35	C24	115.5(5)
C73	C64	1.376(11)	C42	C86	C23	120.8(6)
C18	C9	1.538(9)	C43	C86	C42	118.1(5)

C18	C28	1.540(8)
C43	C57	1.376(9)
C37	C28	1.495(9)
C37	C48	1.419(10)
C37	C49	1.346(12)
C68	C57	1.380(10)
C68	C55	1.378(12)
C45	C59	1.390(8)
C65	C53	1.31(2)
C65	C51	1.36(2)
C48	C62	1.379(10)
C29	C40	1.375(12)
C29	C41	1.428(12)
C29	C19	1.511(7)
C40	C51	1.383(13)
C41	C53	1.378(9)
C59	C47	1.347(10)
C36	C47	1.403(10)
C49	C64	1.408(13)
Na32	Na12[1]	2.758(13)

[1]1-y,1+x-y,+z

C43	C86	C23	121.1(5)
C35	C24	C16	119.4(4)
C36	C24	C35	121.5(5)
C36	C24	C16	119.0(5)
N6	C16	C24	114.8(4)
C62	C73	O81	122.6(7)
C62	C73	C64	121.4(7)
C64	C73	O81	115.8(8)
N7	C18	C9	110.0(4)
N7	C18	C28	112.2(5)
C9	C18	C28	107.5(5)
C86	C23	C15	112.9(4)
C57	C43	C86	121.0(6)
C48	C37	C28	119.7(7)
C49	C37	C28	122.1(7)
C49	C37	C48	118.2(7)
O78	C68	C57	118.9(8)
O78	C68	C55	121.5(6)
C55	C68	C57	119.6(6)
O58	C45	C59	109.9(5)
C35	C45	C35	C45
C35	C45	C59	122.5(5)
O21	C8	O22	119.990(15)
O2	C8	O22	119.988(14)
O21	C8	O2	119.987(15)
O3	C9	O20	123.9(6)
O3	C9	C18	119.5(5)
O20	C9	C18	116.5(5)

C37	C28	C18	112.7(5)
C53	C65	O76	111.0(17)
C53	C65	C51	122.5(8)
C51	C65	O76	126.4(16)
C62	C48	C37	120.0(7)
C40	C29	C41	118.2(6)
C40	C29	C19	122.9(7)
C41	C29	C19	118.8(6)
C43	C57	C68	120.4(7)
C68	C55	C42	120.1(6)
C29	C40	C51	124.7(12)
C53	C41	C29	114.4(9)
C73	C62	C48	120.0(6)
O58	C71	O72	109.4(6)
O72	C59	C45	108.8(6)
C47	C59	O72	129.0(6)
C47	C59	C45	122.3(6)
C65	C53	O67	110.9(9)
C65	C53	C41	125.4(11)
C41	C53	O67	123.7(12)
C65	C51	C40	114.7(12)
N7	C19	C29	113.2(4)
C24	C36	C47	121.0(6)
C37	C49	C64	122.6(7)
C59	C47	C36	117.3(6)
C73	C64	C49	117.6(8)
O76	C77	O67	110.1(10)
O20	Na32	Na12[1]	96.5(4)

O4	Na12	Na32[2]	119.0(4)

[1]1-y,1+x-y,+z; [2]+y-x,1-x,+z

Table **S5**. Hydrogen Bonds features developed in **1Ni-SC**, bond lengths in Å and angles in (º).

**D--H···A**	**D—H (Å)**	**H···A (Å)**	**D···A (Å)**	**D--H···A (°)**
O4--H4B···O2 (1)	0.895	1.802	2.619(5)	150.7
C19--H19A···O2 (1)	0.990	2.701	3.403(4)	128.2
O78--H78···O20 (2)	0.840	1.857	2.675(8)	166.2
C62 --H62···O21 (3)	0.949	2.589	3.233(9)	125.5
O81--H81···O21 (3)	0.839	1.819	2.634(7)	163.5
C16--H16B···O4	0.990	2.55	2.973(6)	105.7
C18 --H18···O78 (4)	1.000	2.558	3.218(9)	123.3
C23 --H23A···O3	0.990	2.493	3.280(7)	136.3
C28 --H28A···O5	0.990	2.473	3.283(7)	139.4
C28 --H28B···O58 (4)	0.990	2.481	3.464(10)	169.0
C48 --H48···O78 (4)	0.950	2.531	3.420(11)	154.4

[1]+Y-X,1-X,+Z; [2]1/3+Y-X,5/3-X,-1/3+Z; [3]2/3+Y-X,4/3-X,1/3+Z; [4]5/3–Y,4/3+X–y,1/3+Z

Table S6: Bond Lengths in Å for **2Ni-SC**.

**Atom**	**Atom**	**Length/Å**
Ni1	O5	2.043(3)
Ni1	N1	2.107(3)
Ni1	N2	2.145(3)
Ni1	O2	2.032(3)
Ni1	O9	2.045(3)
Ni1	O10'	2.026(7)

Ni1	O10	2.168(7)
O5	C18	1.271(4)
O6	C18	1.246(5)
O3	C14	1.387(5)
O3	C17	1.433(6)
O4	C13	1.388(5)
O4	C17	1.434(5)
N1	C2	1.477(5)
N1	C10	1.485(5)
N2	C19	1.481(5)
N2	C27	1.488(5)
O7	C31	1.384(5)
O7	C34	1.429(6)
O8	C30	1.386(5)
O8	C34	1.421(5)
O2	C1	1.263(6)
C28	C33	1.389(5)
C28	C29	1.414(5)
C28	C27	1.507(5)
C31	C32	1.361(6)
C31	C30	1.381(6)
C33	C32	1.402(5)
C13	C12	1.360(5)
C13	C14	1.380(6)

Table **S7**. Bond Angles in (°) for **2Ni-SC**

Atom	Atom	Atom	Angle/˚

O5	Ni1	N1	89.37(11)
O5	Ni1	N2	79.55(11)
O5	Ni1	O9	170.28(13)
O5	Ni1	O10	83.2(2)
N1	Ni1	N2	167.75(12)
N1	Ni1	O10	82.3(2)
N2	Ni1	O10	101.4(2)
O2	Ni1	O5	95.45(13)
O2	Ni1	N1	80.26(12)
O2	Ni1	N2	95.48(12)
O2	Ni1	O9	90.03(18)
O2	Ni1	O10	162.5(2)
O9	Ni1	N1	99.49(12)
O9	Ni1	N2	91.96(12)
O9	Ni1	O10	94.0(3)
O10'	Ni1	O5	100.9(2)
O10'	Ni1	N1	89.3(2)
O10'	Ni1	N2	97.8(2)
O10'	Ni1	O2	160.5(2)
O10'	Ni1	O9	75.4(3)
C18	O5	Ni1	114.4(2)
C14	O3	C17	103.8(3)
C13	O4	C17	104.1(3)
C2	N1	Ni1	108.9(2)
C2	N1	C10	113.0(3)
C10	N1	Ni1	113.7(2)

C19	N2	Ni1	105.3(2)
C19	N2	C27	113.0(3)
C27	N2	Ni1	109.7(2)
C31	O7	C34	105.0(3)
C30	O8	C34	105.4(3)
C1	O2	Ni1	115.7(3)
C33	C28	C29	119.3(3)
C33	C28	C27	119.1(3)
C29	C28	C27	121.6(3)
C32	C31	O7	128.8(4)
C32	C31	C30	121.5(4)
C30	C31	O7	109.7(4)
C28	C33	C32	122.4(4)
C12	C13	O4	128.0(4)
C12	C13	C14	122.8(4)
C14	C13	O4	109.2(3)
C12	C11	C10	118.6(3)
C16	C11	C12	120.1(4)
C16	C11	C10	121.2(3)
C14	C15	C16	116.4(4)

Table **S8**. Hydrogen Bonds for **2Ni-SC**.

**D**	**H**	**A**	**d(D-H)/Å**	**d(H-A)/Å**	**d(D-A)/Å**	**D-H-A/°**
N2	H2	$O1^{1}$	1.001	2.177	2.980(7)	136.3
O9	H9A	$O1^{1}$	0.928	2.049	2.903(7)	152.2
O9	H9B	$O6^{1}$	0.928	1.825	2.715(4)	159.8

O10'	H10A	O13[2]	0.873	2.027	2.863(9)	160.0
O13	H13A	O12[3]	0.870	2.318	2.938(9)	128.3
O13	H13B	O6	0.870	1.922	2.758(5)	160.9
O12	H12A	O2	0.868	2.024	2.882(6)	169.7
O12	H12B	O5[1]	0.871	2.094	2.955(5)	169.7
N2	H2	O11	1.00	2.18	2.980(7)	136.3

[1]-1/2+X,3/2-Y,1-Z; [2]+X,+Y,-1+Z; [3]1/2+X,3/2-Y,2-Z

Table **S9**. Atomic Occupancy for **2Ni-SC**.

**Atom**	***Occupancy***
O10'	0.49
O1	0.48
H10E	0.51
H10A	0.49
O1'	0.52
H10F	0.51
H10B	0.49
O10	0.51

Powder XRD studies

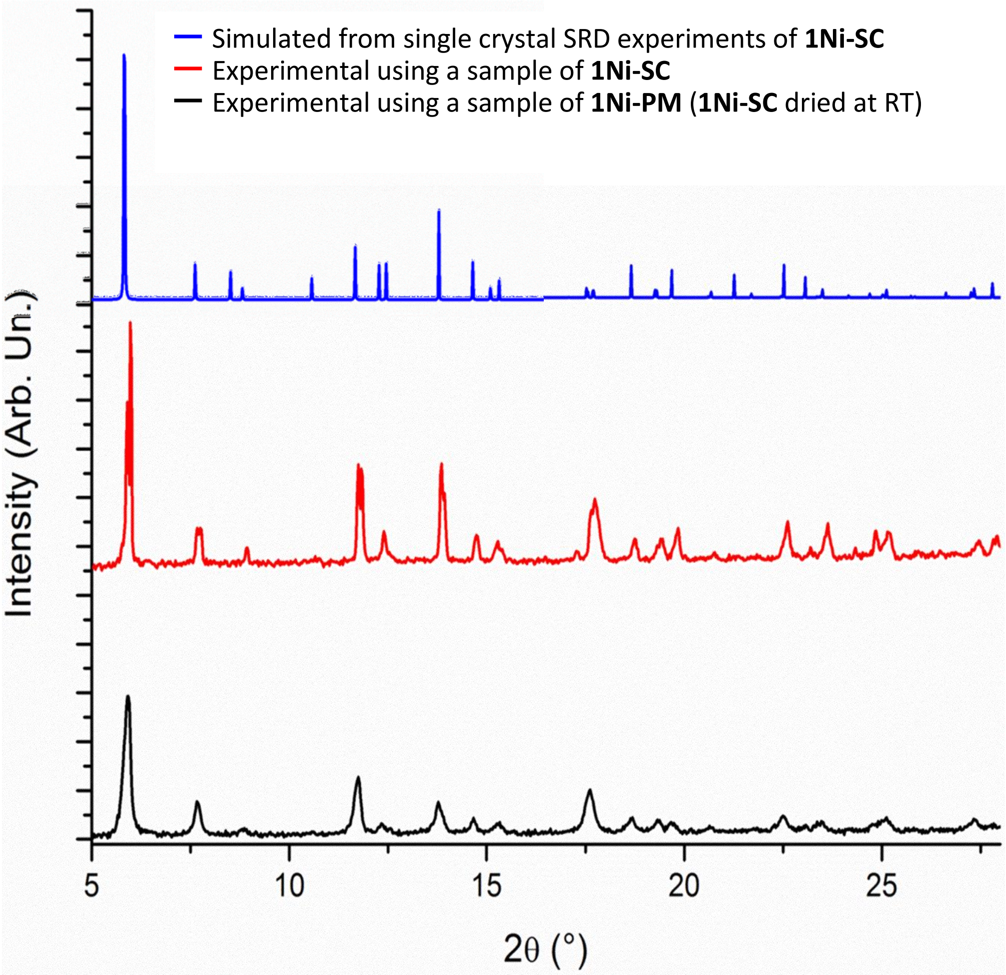

Figure S24 pXRD diffraction patterns for **1Ni**: Simulated from single crystal structure (blue), obtained for sample of single crystals freshly obtained from the mother liquor (red) and polycrystalline sample obtained after drying the single crystals at room temperature (black). The broadening of the signals in the polycrystalline material could be associated with the partially loss of crystallinity. Nevertheless, all three diffractograms are in good agreement.

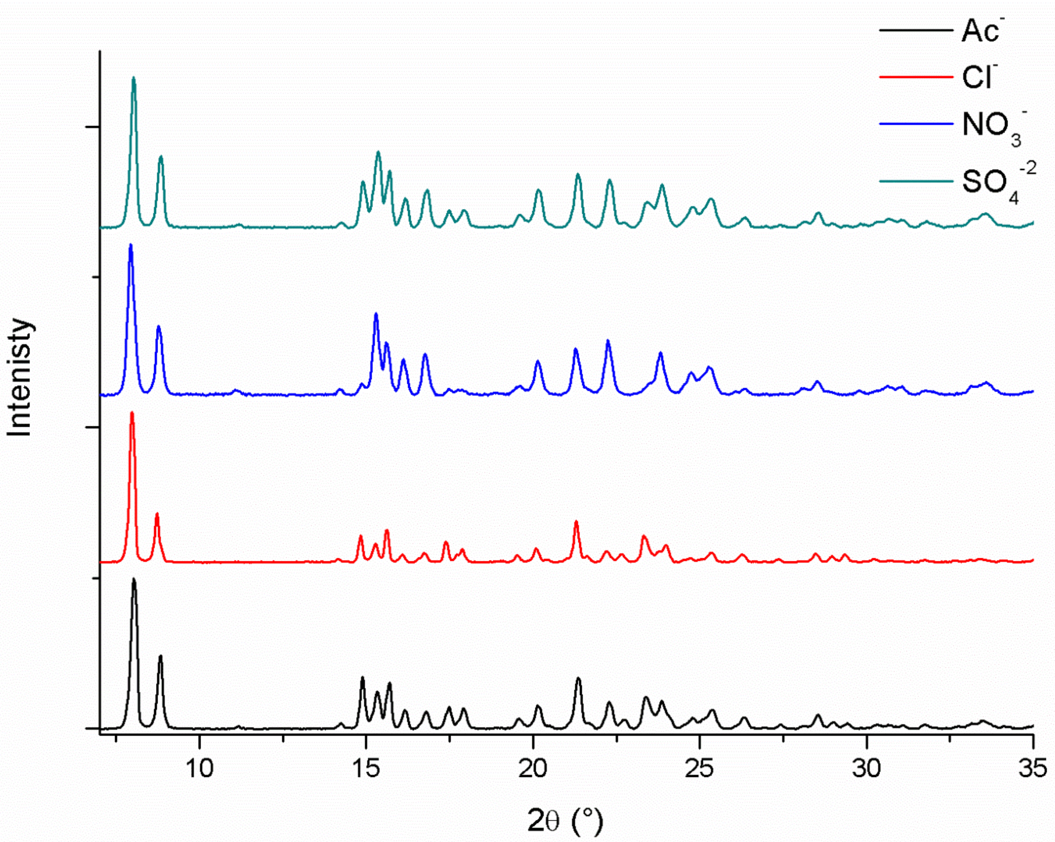

Figure S25 X-ray diffraction patterns for **2Ni-CA** using different Nickel (II) salts for the synthesis.

Additional structural analysis

The molecular structure of **1Ni-SC** was evaluated along with the data deposited at the CSD and it was confirmed that the structural parameters of all fragments are in agreement with average values of the most relevant bond lengths and angles.

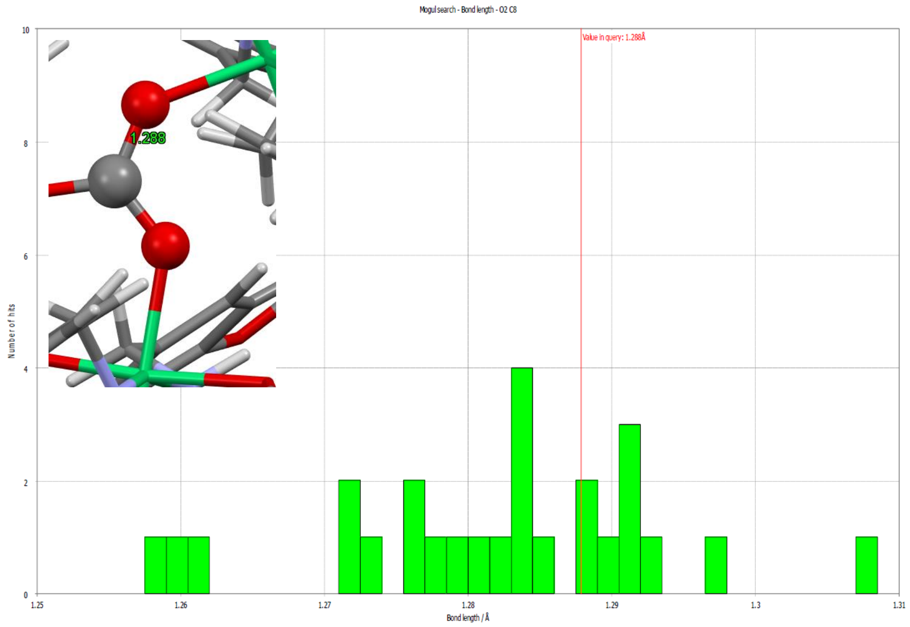

Figure S26 Statistical evaluation for carbonate angles in **1Ni** complex.

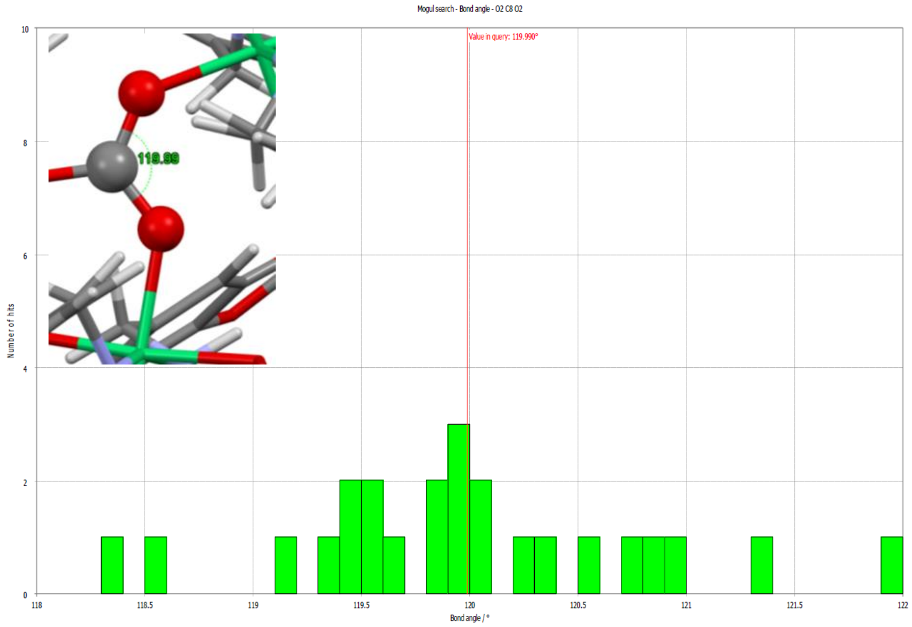

Figure S27 Statistical evaluation of the C8-O2 bond length of the carbonate in **1Ni** complex.

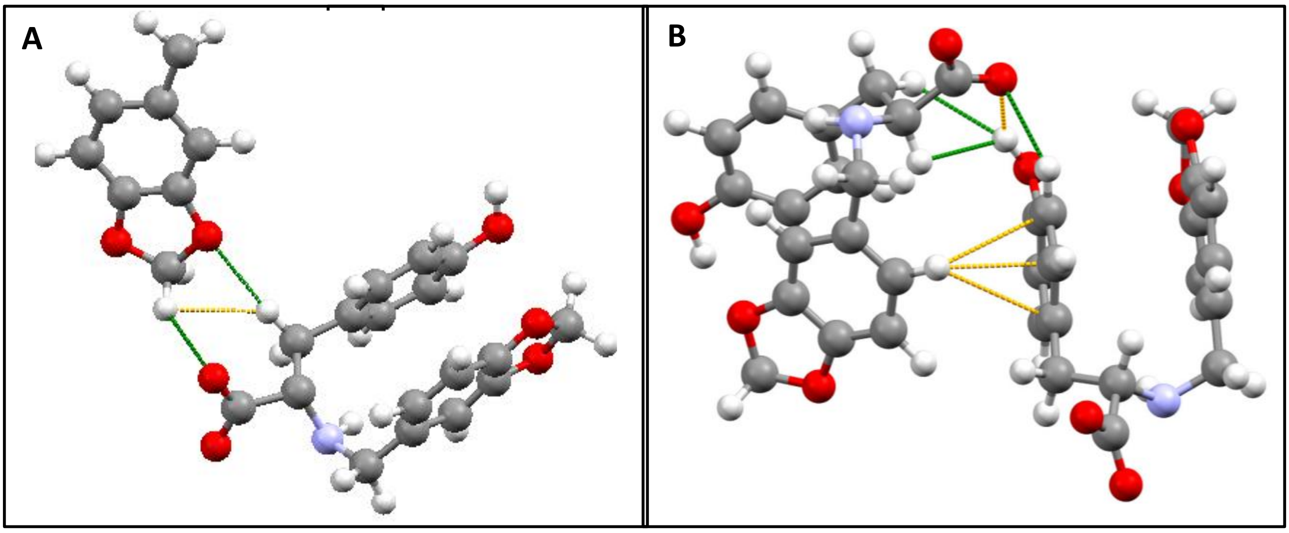

Figure S28 Intermolecular short contacts developed in **1Ni-SC** (C–H···O, C–O···H–C, C–H···π). For clarity only ligand **1** (completely or partially) is shown.

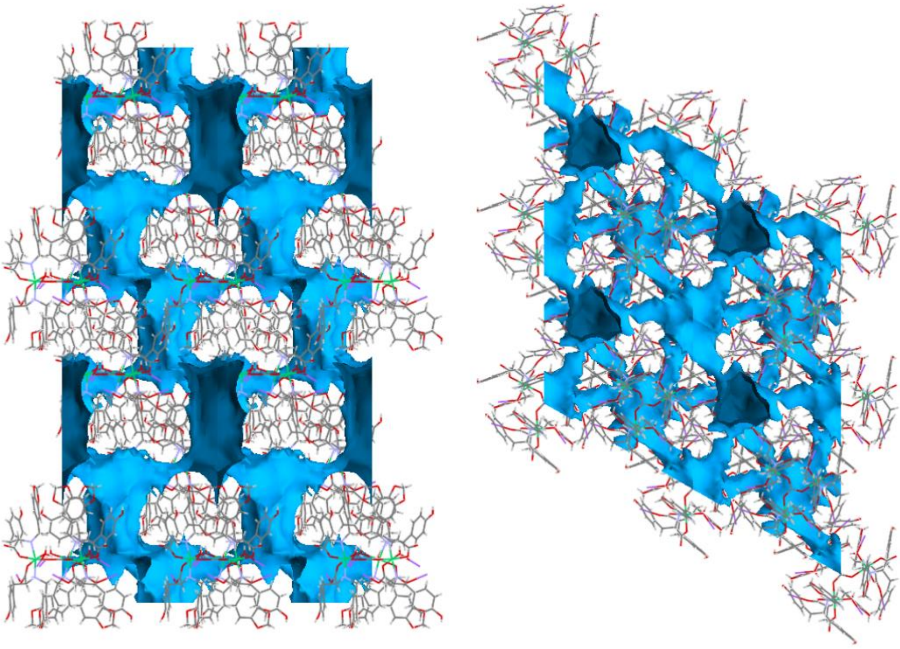

Figure S29 Accessible voids observed the extended structure of **1Ni-SC** in plotted in light-blue. Left, view along crystallographic plane *ac* and right, along plane *ab*.

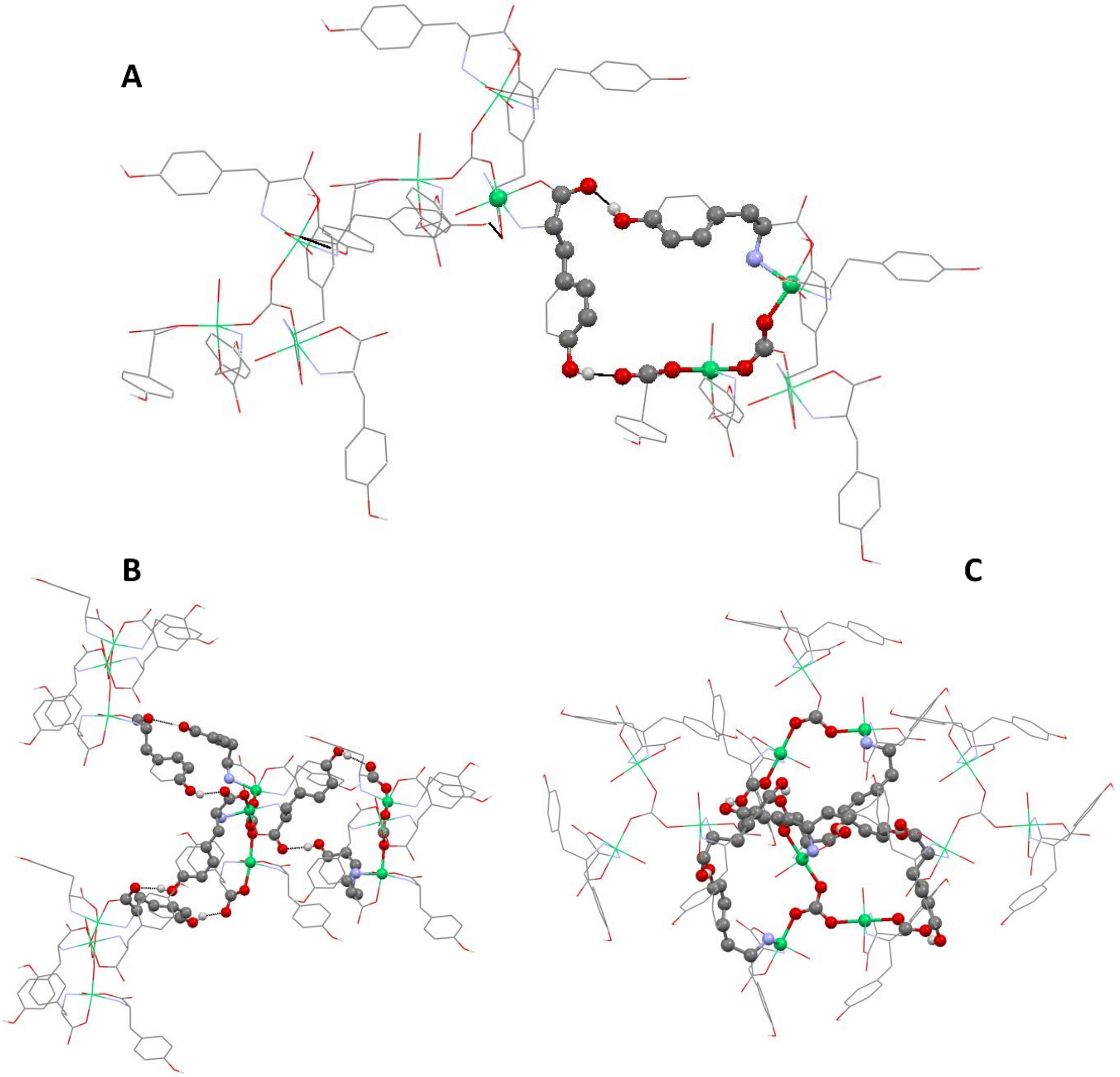

Figure S30 $R_2^2(27)$ Graph Sets (in balls) described in **1Ni-SC**. **A**: Ring resulted when one of the ligands interact with two ones belonging to the same trinuclear complex; **B&C**: the ring described in **A** together with the sets obtained when the other ligand of the same center interacts with the corresponding ligands. **B** view along crystallographic plane *ac* and **C** along ab. In the images, the counterions, the piperonal moieties and H atoms which do not participate of the short contacts are omitted for clarity.

The ligands in the structure of **2Ni**, one of them showed a disordered carboxylate moiety and besides, the relative location of the aromatic rings respect to each other is also different in each ligand. In one of the ligands the rings are displayed in a parallel fashion as it was observed for the structure of complex **1Ni**, giving place to a long distance π-π interaction (distance between centroids 3.825 Å), but the other has the piperonal moiety a bit away from the expected location. The angle between the planes described by the rings shows a torsion of 43.26°. A possible explanation for that could be associated with the effect of the intermolecular interactions developed by the DMF located in the proximity of the second ligand. It is probable that the H-bonds established by this molecule and the carboxylate and amino groups would balance the distortion generated in the rings and the absence of any π-interaction (Figure S31).

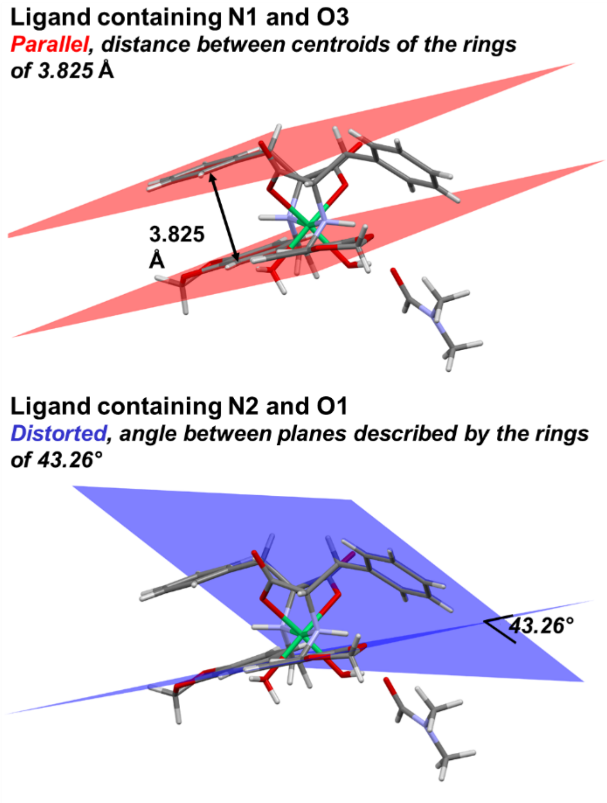

Figure S31 Conformation of the ligands in **2Ni-SC**.

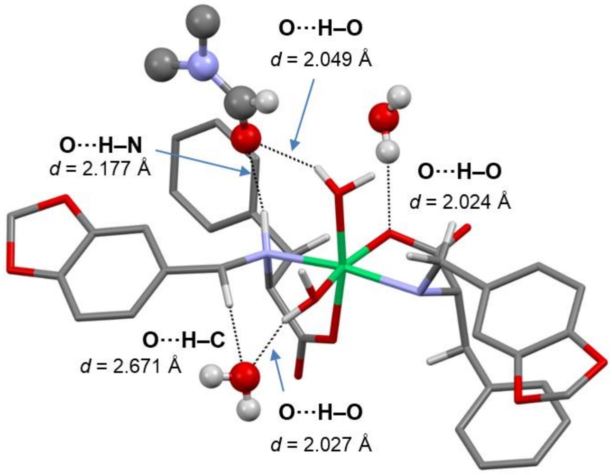

Figure S32 **2Ni-SC** showing two water molecules and one DMF included in the structure. H-bond interactions are also indicated. Solvent molecules are indicated in isotropic balls&sticks style.

Supramolecular structure of **2Ni-SC** is constructed by an infinite linear chain described by consecutive units of the complex along crystallographic axis *c*, which interacts with a zig zag arrangement extended along crystallographic axis *a* and also, by C–H···O contacts involving the piperonal moieties which are displayed along crystallographic axis *b*.

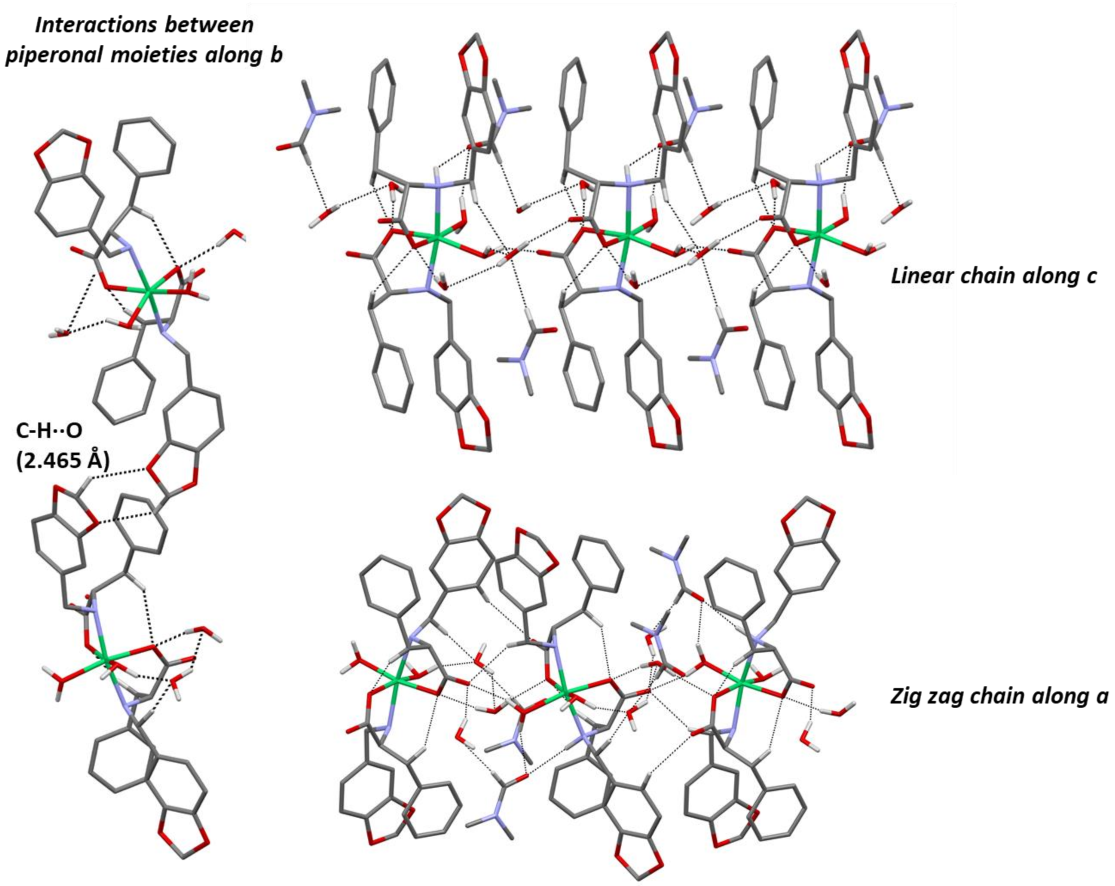

Figure S33 Details of the H-bonds exhibited in **2Ni-SC.**

4. Supplementary Magnetic Measurements

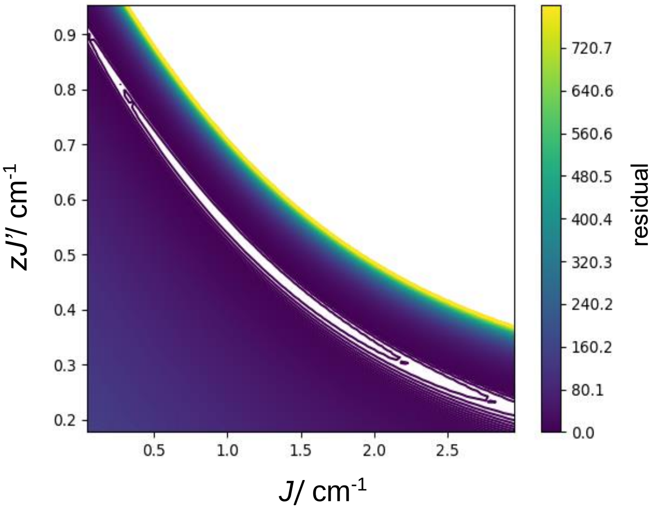

Figure S34 Fitting error surface (Hamiltonian Eq. 1) with *g* and *D* parameters fixed at best values showing the strong *J* / *zJ*' parameters correlation.

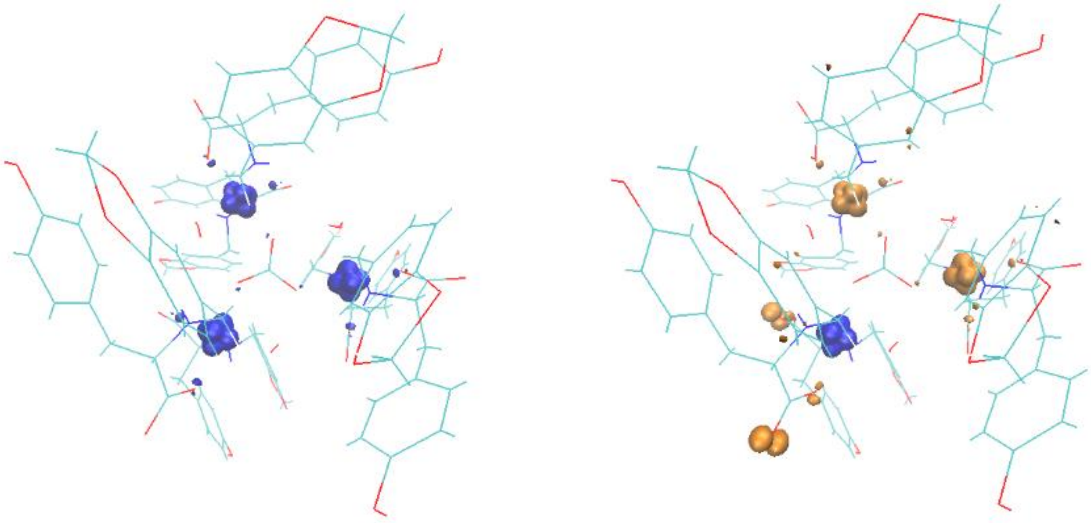

Figure S35 Isosurfaces (0.02 a.u.) corresponding to the spin density arising from the high spin state (left) and broken-symmetry state (right).

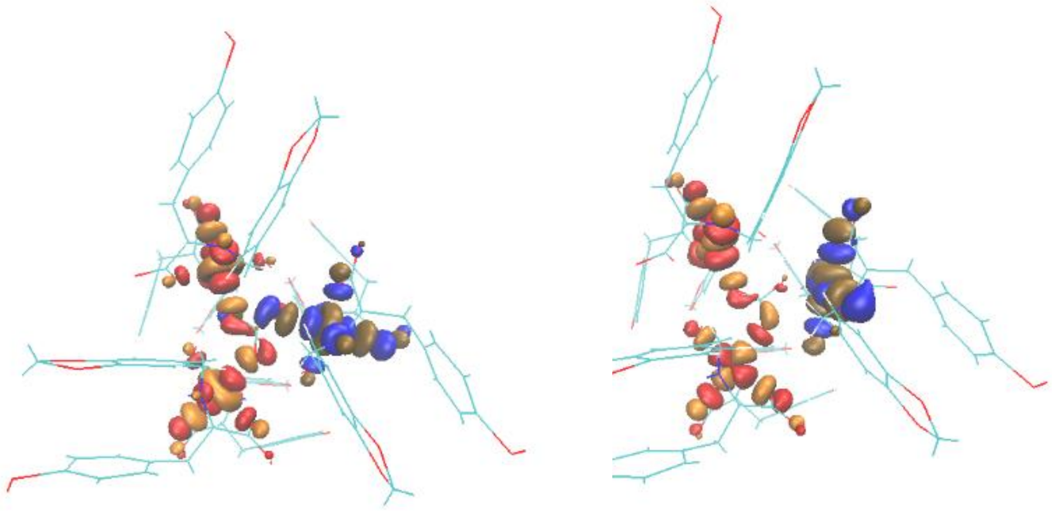

***S* = 0.01421** **S = 0.00172**

Figure S36 Isosurfaces (0.02 a.u.) corresponding to the α-β magnetic orbitals pairs after a corresponding orbital transformation (COT) together with the overlap integrals

5. Supplementary Information References

S1 CAZTIJ and CAZTOP: Muche, S. & Hołyńska, M. (2017). *J. Mol. Struct.* **1142**, 168–174.

S2 FEZKIH01: Tong, J., Zhao, L., Li, H., Wu, C., Han, X., Wang, J. & Liu, H. (2018). *Tetrahedron* **74**, 3755–3760.

S3 FEZKIH: Tong, J., Zhao, L., Li, H., Wu, C., Han, X., Wang, J. & Liu, H. (2018). Private communication (refcode FEZKIH). CSD Comm.

S4 ISUGEJT: New, S. Y., Wu, X., Bai, S.-Q., Koh, L. L., Hor, T. S. A. & Xue, F. (2011). *Cryst. Eng. Comm.* **13**, 4228.

S5 CAJYAQ: Huang, G., Zhang, X., Fan, Y., Bi, C., Yan, X., Zhang, Z. & Zhang, N. (2013). *Bull. Korean Chem. Soc*. **34**, 2889–2894.

S6 COHMUJ: Zhang, Y., Li, H., Ou-Yang, M. & Zhang, H. (2012). *Huaxue Yanjiu Yu Yingyong (Chin.) (Chem. Res. Appln.)* **24**, 1592–1595.

S7 IDOYOQ: Pei, Y. & Wang, L. (2006). *Acta Cryst.* E**62**, m1668–m1670.

S8 LTYRNI: Hamalainen, R., Ahlgren, M., Turpeinen, U. & Raikas, T. (1978). *Cryst. Struct. Comm*. **7**, 379–384.

S9 LTYRNI01: Gao, C., Ma, X., Kou, Y., Li, D., Feng, L., Tian, J. & Yan, S. (2009). *Nankai Daxue Xuebao, Ziran Kexueban (Chin.) (Acta Scient. Nat. Univ. Nankaiensis)* **42**, 103–104.

S10 NISBOI: Wojciechowska, A., Gągor, A., Duczmal, M., Staszak Z. & Ozarowski, A. (2013). *Inorg. Chem.* **52**, 4360–4371.

S11 RAPZAL: Wojciechowska, A., Daszkiewicz, M., Staszak, Z., Trusz-Zdybek, A., Bieńko, A. & Ozarowski, A. (2011). *Inorg. Chem.* **50**, 11532–11542.

S12 TYRANI: Hamalainen, R., Lajunen, K. & Valkonen, J. (1977). *Finn. Chem. Lett*. ***4-5***, 108–112.

S13 DIHDIJ: Prior, T. J., Rujiwatra, A. & Tapala, W. (2013). *J. Chem. Cryst.* **43**, 299–305.

S14 IDIQET: Tapala, W., Prior, T. J. & Rujiwatra, A. (2013). *Acta Cryst.* E**69**, m286–m287.

S15 OGULAF: Wojciechowska, A., Janczak, J., Staszak, Z., Duczmal, M., Zierkiewicz, W., Tokar, J. & Ozarowski, A. (2015). *New J. Chem.* **39**, 6813–6822.

S16 Groom, C. R., Bruno, I. J., Lightfoot, M. P. & Ward, S. C. (2016). *Acta Cryst.* B**72**, 171–179.

S17 HUHKEA: Cai, J., Hu, X., Bernal, I. & Ji, L. N. (2002). *Polyhedron*. **21**, 817–823.

S18 IYASAC: Wang, L., Cai, J., Mao, Z.-W., Feng, X.-L. & Huang, J.-W. (2004). *Trans. Metal Chem.* **29**, 411–418.

S19 KOBJOB, KOBJUH, KOBKES, KOFNID and KOFNOJ: Ou, G. C., Jiang, L., Feng, X. L. & Lu, T. B. (2008). *Inorg. Chem.* **47**, 2710–2718.

S20 YATYID: Ghorbanloo, M., Shahbakhsh, N. & Choquesillo-Lazarte, D. (2012) *Acta Cryst.* E**68**, m446–m446.

S21 HISMEC and YISRAU: Mustapha, A., Busch, K., Patykiewicz, M., Apedaile, A., Reglinski, J., Kennedy, A. R. & Prior, T. J. (2008). *Polyhedron*, **27**, 868–878.

S22 OHARUL: Anderson, J. C., Blake, A. J., Moreno, R. B., Raynel, G. & van Slageren, J. (2009). *Dalton Trans.*, 9153–9156.

S23 LONFOK: Miyamoto, K., Horn, E. & Fukuda, Y. (2008). *Z. Krist. NCS.* **223**, 523–528.

S24 GOHBUB: Mukherjee, P., Drew, M. G. B., Estrader, M. & Ghosh, A. (2008). *Inorg. Chem.* **47**, 7784–7791.

S25 HOSDOI: Escuer, A., el Fallah, M. S., Kumar, S. B., Mautner, F. & Vicente, R. (1998). *Polyhedron*, **18**, 377–381.

S26 IBOLUH: Alcock, N. W., Moore, P., Clase, H. J. & Rawle, S. (2004). Private communication (refcode IBOLUH). CSD Comm.

S27 IBOLUH01: Fu, H., Chen, W. L., Fu, D. G., Tong, M. L., Chen, X. M., Ji, L. N. & Mao, Z. W. (2004). *Inorg. Chem. Comm.* **7**, 1285–1288.

S28 TAVQOW: Escuer, A., Vicente, R., Kumar, S. B., Solans, X., Font-Bardía, M. & Caneschi, A. (1996). *Inorg. Chem.* **35**, 3094–3098.

S29 Oxford Diffraction/Agilent Technologies UK Ltd.

S30 Blessing, R. H. (1995). *Acta Cryst.* A**51**, 33–38.

S31 Coppens, P., Leiserowitz, L. & Rabinovich, D. (1965). *Acta Cryst.* **18**, 1035–1038.
